# Multi-Omics Reveals Disrupted Immunometabolic Homeostasis and Oxidative Stress in Adipose Tissue of Dairy Cows with Subclinical Ketosis: A Sphingolipid-Centric Perspective

**DOI:** 10.3390/antiox13050614

**Published:** 2024-05-17

**Authors:** Huiying Zhao, Liuxue Li, Jian Tan, Ying Wang, Ao Zhang, Yuchao Zhao, Linshu Jiang

**Affiliations:** Beijing Key Laboratory of Dairy Cow Nutrition, College of Animal Science and Technology, Beijing University of Agriculture, Beijing 102206, China; 202130312010@bua.edu.cn (H.Z.); 202130312014@bua.edu.cn (L.L.); 202230312013@bua.edu.cn (J.T.); 202230321112@bua.edu.cn (Y.W.); 202330321119@bua.edu.cn (A.Z.)

**Keywords:** adiponectin, ceramide, dairy cows, insulin resistance, oxidative stress, subclinical ketosis

## Abstract

Ketosis, especially its subclinical form, is frequently observed in high-yielding dairy cows and is linked to various diseases during the transition period. Although adipose tissue plays a significant role in the development of metabolic disorders, its exact impact on the emergence of subclinical ketosis (SCK) is still poorly understood. The objectives of this study were to characterize and compare the profiling of transcriptome and lipidome of blood and adipose tissue between SCK and healthy cows and investigate the potential correlation between metabolic disorders and lipid metabolism. We obtained blood and adipose tissue samples from healthy cows (CON, n = 8, β-hydroxybutyric acid concentration < 1.2 mmol/L) and subclinical ketotic cows (SCK, n = 8, β-hydroxybutyric acid concentration = 1.2–3.0 mmol/L) for analyzing biochemical parameters, transcriptome, and lipidome. We found that serum levels of nonesterified fatty acids, malonaldehyde, serum amyloid A protein, IL-1β, and IL-6 were higher in SCK cows than in CON cows. Levels of adiponectin and total antioxidant capacity were higher in serum and adipose tissue from SCK cows than in CON cows. The top enriched pathways in whole blood and adipose tissue were associated with immune and inflammatory responses and sphingolipid metabolism, respectively. The accumulation of ceramide and sphingomyelin in adipose tissue was paralleled by an increase in genes related to ceramide biosynthesis, lipolysis, and inflammation and a decrease in genes related to ceramide catabolism, lipogenesis, adiponectin production, and antioxidant enzyme systems. Increased ceramide concentrations in blood and adipose tissue correlated with reduced insulin sensitivity. The current results indicate that the lipid profile of blood and adipose tissue is altered with SCK and that certain ceramide species correlate with metabolic health. Our research suggests that disruptions in ceramide metabolism could be crucial in the progression of SCK, exacerbating conditions such as insulin resistance, increased lipolysis, inflammation, and oxidative stress, providing a potential biomarker of SCK and a novel target for nutritional manipulation and pharmacological therapy.

## 1. Introduction

The excessive energy demands associated with high milk production often result in negative energy balance (NEB) in high-yielding dairy cows in early lactation [1]. This NEB condition leads to a higher release of nonesterified fatty acids (NEFA) and a rise in ketone bodies like β-hydroxybutyric acid (BHBA). Cows that struggle to adjust to this energy deficit are more susceptible to various metabolic disorders [2,3]. Such metabolic problems and diseases may decrease the productive life of the cows, affect their welfare negatively, and lead to higher expenses for veterinary care. Hyperketonemia is one of the most common and economically damaging metabolic disorders occurring during the periparturient period [4]. Subclinical ketosis (SCK) is defined as elevated levels of ketone bodies in the blood without visible symptoms of the disease [5]. It is estimated that up to half of all dairy cows experience a phase of SCK during the initial month of lactation [6]. The economic repercussions and the adverse effects on animal welfare associated with SCK present significant challenges for dairy producers [4,5]. Therefore, understanding SCK’s underlying mechanisms is crucial for its detection, prevention, and timely intervention.

The use of systems biology techniques has been instrumental in advancing our understanding of the metabolic and molecular alterations associated with ketosis. This has been demonstrated through studies on the plasma metabolome [7,8], the hepatic transcriptome and metabolome [9], as well as the milk metabolome and lipidome [10]. Adipose tissue serves two primary functions: it acts as an energy regulator by managing the storage and release of fatty acids and as a crucial endocrine organ by producing hormones and adipokines that influence metabolic processes [11]. In diseases like SCK and displaced abomasum, there is a significant drop in the body’s insulin sensitivity, leading to increased fat breakdown [12,13]. Although adipose tissue plays a vital role in the development of metabolic illnesses, the specifics of how it contributes to SCK’s development are still unclear.

A transcriptomic study on cow fat tissue revealed significant gene expression changes related to nutrient and metabolite utilization, inflammation, immune responses, cell growth, tissue modification, and blood vessel formation during lactation [14]. Mellouk et al. [15] observed that severe NEB in dairy cows leads to significant changes in the gene expression patterns within subcutaneous adipose tissue, particularly affecting lipid metabolism. Furthermore, the proteomic analysis indicated that cows with ketosis in the transition phase exhibit disrupted carbohydrate and fat metabolism and compromised immune responses in their adipose tissue [16]. A recent study revealed that the reduced expression of *NTRK2* in subcutaneous adipose tissue might impair the nervous regulation of lipid metabolism and disrupt the balance between lipogenesis and lipolysis in ketosis dairy cows [17]. Altered lipide metabolism significantly influences the onset of ketosis. Lipidomics emerged as a superior method for analyzing disruptions in lipid homeostasis [18]. Its key benefit over other broad analysis approaches like transcriptomics or proteomics lies in its ability to track lipids, which are the ultimate manifestation of genetic or protein interactions with the environment, essentially mirroring the phenotype [19]. However, research into how SCK correlates with a comprehensive multi-omics analysis in the adipose tissue of dairy cows at the start of lactation is still scarce.

Additionally, there is an effort to create a novel, less invasive, and more precise diagnostic method that can be applied to single animals and entire herds. Blood circulation offers a minimally invasive substitute for tissue biopsies for the molecular characterization of diseases and their risks [20]. Profiling transcript abundance and lipid features in the blood provides a ‘snapshot’ of the complex immune networks and lipid homeostasis that operate throughout the body [21]. We assumed that an imbalance in the lipid homeostasis of adipose tissue can lead to systemic metabolic disorders. A cow with SCK may exhibit alterations in circulating lipid composition, systemic inflammation, and immune responses that can be detected by metabolomics and transcriptomics.

Therefore, we combined transcriptomics and lipidomics data from cows with SCK to enhance the comprehension of their biological state and uncover new information on potential molecular mechanisms, interactions, and biomarkers linked to the association between SCK and lipid metabolism disorders. We hypothesized that the transcriptome and lipidome of blood and adipose tissue of SCK cows would show greater lipolysis, lipid metabolism imbalance, increased inflammatory response, oxidative stress, and exacerbated insulin resistance compared to healthy cows.

## 2. Materials and Methods

### 2.1. Experimental Design, Retrospective Analysis, and Animal Management

This experiment was conducted following the China Laboratory Animal Welfare and Ethics Committee Guidelines at a commercial dairy farm in Beijing, China, milking 1800 cows. The animal use protocol was approved by the Animal Care Committee at the Beijing University of Agriculture (Animal Use Protocol # BUA2022052).

**Figure 1:**
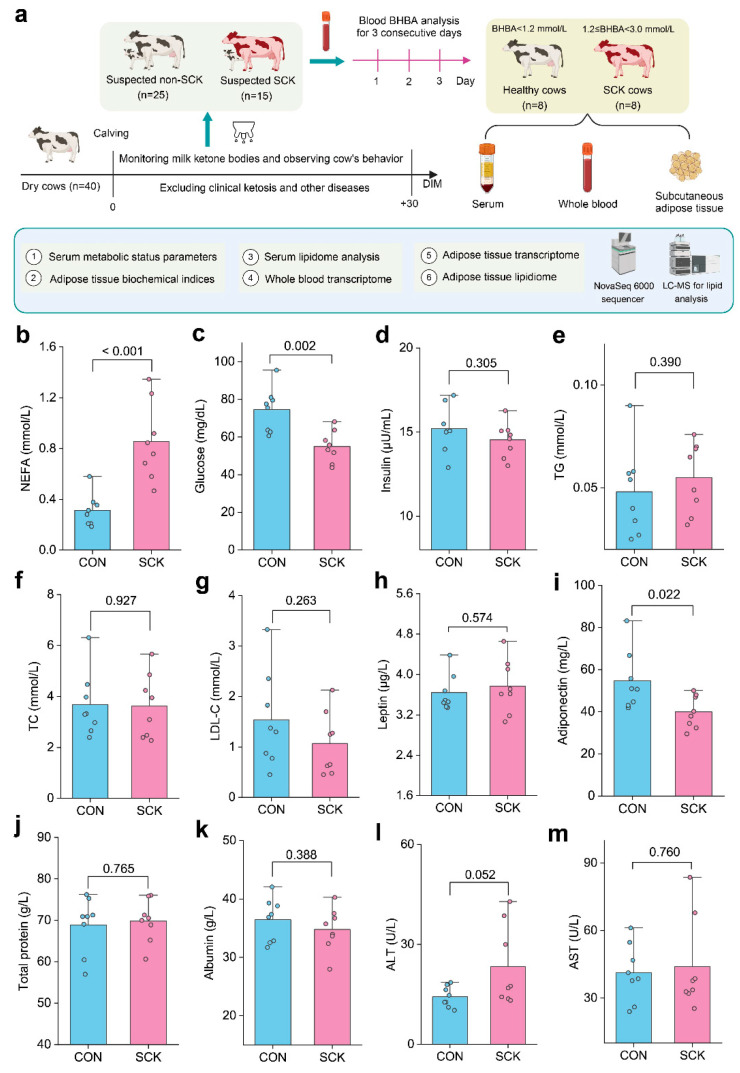
Serum glucose/lipid metabolism and liver function indices. (**a**) Experimental design. (**b**–**h**) Serum glucose and lipid metabolism parameters: (**b**) NEFA; (**c**) glucose; (**d**) insulin; (**e**) TG; (**f**) TC; (**g**) LDL-C; (**h**) leptin; (**i**) adiponectin. (**j**–**m**) Liver function: (**j**) total protein; (**k**) albumin; (**l**) ALT; (**m**) AST. CON: healthy cows; SCK: subclinical ketosis; NEFA: nonesterified fatty acids; TG: triglyceride; TC: total cholesterol; LDL-C: low-density lipoprotein cholesterol; ALT: alanine aminotransferase; AST: aspartate aminotransferase.

A total of 40 multiparous dry cows were randomly selected 40 days before calving. All cows were housed in a freestall barn (with rubber beds and rice hull bedding) with the Roughage Intake Control system (Insentec) from 7 d prepartum to 30 d postpartum. Individual dry matter intake (DMI) was recorded daily by the Roughage Intake Control System, which can identify the cow ID before opening the trough and measure the feed weight before and after eating. Body weight (BW) was measured, and body condition score (BCS) was assessed 4 h after morning feeding at 1100 h weekly. On calving day, cows were weighed and their BCS was assessed after the collection of colostrum. The BCS of cows was assessed by the same two personnel using a standard 5-point scale (1 = thin to 5 = fat) in 0.25-unit increments. Twenty-five dairy cows were categorized as suspected non-SCK and 15 cows were categorized as suspected SCK based on nitroprusside tests for ketone bodies in milk. Blood concentrations of BHBA were measured for three consecutive days to choose healthy and SCK cows. Lastly, 10 dairy cows with serum 1.2 mmol/L < BHBA < 3.0 mmol/L for all 3 d were classified as SCK cows, and 15 cows with serum BHBA < 1.2 mmol/L for all 3 d were classified as healthy control group (CON). Finally, 8 cows were selected randomly in each group for sample analyses (Figure 1a). There was no diagnosis of clinical ketosis (CK; BHBA > 3.0 mmol/L) among the cows during the screening process. The selected cows did not display any clinical signs of disease after calving, including metritis, lameness, mastitis, displaced abomasum, and dyspepsia. Cows were milked 3 times each day after calving at 0700, 1300, and 2100 h, and milk production was recorded daily. Table S1 presents a basic description of the 16 selected cows used in the study.

All cows had free access to fresh water. All cows followed a uniform close-up diet and were fed the same lactation diet from parturition to 30 days postpartum. The diets before and after parturition were formulated to meet the guidelines of the National Research Council [22]. At the beginning of the study, total mixed ration (TMR) samples for both the close-up and lactation diets were assessed for dry matter (DM; AOAC, ID 930.15) [23], crude protein (CP; AOAC, 984.13) [23], ether extract (EE; AOAC, ID 920.39)[23], ash (AOAC, ID 942.05) [23], and neutral detergent fiber (NDF) [24]. During the far-off and early lactation period, the dry cow and lactating cow TMR was delivered twice daily at 0730 and 1400 h. During the close-up period, the prepartum TMR was delivered once daily at 1400 h. Details on the ingredient and chemical composition of the TMR are provided in Table S2.

### 2.2. Sampling

Blood samples were drawn from the tail veins into tubes with a coagulation agent (BD Vacutainer, Preanalytical Solutions, Franklin Lakes, NJ, USA) for three consecutive days, as mentioned in Section 2.1. These samples were then centrifuged at 2000× *g* at 4 °C for 20 min to collect the serum. The serum was immediately frozen and stored at −80 °C for later analysis. Additionally, 3 mL of blood were collected into tubes with 6 mL of an RNA preservation solution (Tempus Blood RNA Tube, Applied Biosystems, Waltham, MA, USA) and were quickly frozen in liquid nitrogen. The collection over three days aimed to identify cows with SCK, and all samples were used for BHBA analysis using a commercial kit (Shanghai Acmec Biochemical Technology Co., Ltd., Shanghai, China). Only serum from the first day was used for other biochemical parameter analyses in both the SCK and CON groups.

Subcutaneous adipose tissue (SCAT) samples were collected from the right side of selected cows on the final day of the blood sample collection. Briefly, the area was numbed with 15 mL of 2% lidocaine hydrochloride and sterilized using iodine and alcohol. A 2 cm vertical incision was made in the skin, and approximately 3 g of tissue was excised. The samples were then cleaned of any connective tissue and washed in saline to minimize blood residue. For analysis, the samples were divided into three aliquots, placed in tubes, flash-frozen in liquid nitrogen, and stored at −80 °C for later transcriptome and lipidome analyses and to measure biochemical parameters.

### 2.3. Serum Metabolic Status Parameters Analysis

Serum samples were determined for metabolic biomarkers of glucose and lipid metabolism [i.e., NEFA, glucose, insulin, triglyceride, total cholesterol, low-density lipoprotein cholesterol (LDL-C), leptin, adiponectin], liver function indices [i.e., aspartate aminotransferase (AST), alanine aminotransferase (ALT), total protein, albumin], endotoxin [i.e., lipopolysaccharide (LPS)], oxidative status [i.e., superoxide dismutase (SOD), glutathione peroxidase (GSH-Px), total antioxidant capacity (T-AOC), malonaldehyde (MDA)], and inflammatory factor [i.e., C-reactive protein (CRP), haptoglobin (Hp), serum amyloid A protein (SAA), IL-1β, IL-2, IL-6, IL-10, and tumor necrosis factor α (TNF-α)]. Serum parameters were analyzed with commercially available kits according to manufacturers’ instructions (NEFA, BHBA, glucose, insulin, triglyceride, total cholesterol, LDL-C, leptin, LPS, CRP, L-1β, IL-2, IL-6, IL-10, and TNF-α: Nanjing Jiancheng Bioengineering Institute, Nanjing, China; adiponectin, SOD, GSH-Px, T-AOC, and MDA: Beijing Solarbio Science & Technology Co., Ltd., Beijing, China; AST, ALT, total protein, and albumin: Shanghai Acmec Biochemical Technology Co., Ltd., Shanghai, China). Coefficients of variation within and between assays were in all cases lower than 10%. The revised quantitative insulin sensitivity check index (RQUICKI) is determined using the formula: RQUICKI = 1/[log(serum glucose in mg/dL) + log(serum insulin in µU/mL) + log(serum NEFA in mmol/L)] [25]. A lower value of the RQUICKI index suggests a reduction in insulin sensitivity.

### 2.4. Biochemical Parameters Analysis of Subcutaneous Adipose Tissue

Protein from adipose tissue was isolated using radio-immunoprecipitation assay buffer (Thermo Scientific, Dreieich, Germany) enhanced with a protease inhibitor mix (Roche, Indianapolis, IN, USA) and measured via the BCA Assay (Thermo). Levels of pro-inflammatory cytokines in adipose tissue (IL-1β, IL-6, TNF-α), oxidative markers [total antioxidant capacity (T-AOC) and total superoxide dismutase (SOD)], and adiponectin were determined using ELISA kits from the Nanjing Jiancheng Bioengineering Institute (Nanjing, China).

### 2.5. Whole Blood and Adipose Tissue RNA-Seq Data Analysis

Adipose tissue samples (20 mg) were thoroughly homogenized using a TissueLyser II device (Qiagen, Germantown, MD, USA) with 600 µL of Buffer RLT and a 5 mm stainless steel bead for total RNA extraction, following the NucleoSpin RNA II kit (Macherey-Nagel, Düren, Germany) on-column purification method. For whole blood samples (containing leukocytes), the Tempus Spin RNA Isolation Kits (Thermo Fisher) were used as per the provided guidelines. RNA quality and concentration were assessed using a NanoDrop 2000 (Thermo Fisher Scientific, Waltham, MA, USA) and an Agilent 2100 Bioanalyzer (Agilent Technologies, Santa Clara, CA, USA), with RNA integrity confirmed through 2% agarose gel electrophoresis. The average RNA concentration, A260:A280 optical density ratios, and RNA integrity number (RIN) were reported as 0.18 ± 0.07 µg/µL, 2.00 ± 0.04, and 7.7 ± 0.5, respectively (mean ± SD, n = 16). cDNA synthesis was performed using 1 μg of RNA with the High-Capacity cDNA Reverse Transcription Kit (Applied BioSystems). RNA sequencing libraries for adipose tissue and whole blood were prepared using the Illumina TruSeq Stranded Total RNA Library Prep Ribo-Zero Gold kit (Illumina Inc., San Diego, CA, USA) and sequenced on an Illumina NovaSeq 6000 system (2 × 150 bp). The generated FASTQ files were uploaded to the Sequence Read Archive Database at the National Center for Biotechnology Information (PRJNA1076465 and PRJNA1074691).

The initial quality check of raw sequencing data (fastq format) was conducted using FastQC (v0.11.5), followed by the removal of reads of low quality (mean Q-score < 20) or shorter than 30 base pairs, and the elimination of adapter sequences with TrimGalore (v0.5.0). Subsequently, the cleaned reads were aligned to the *Bos taurus* reference genome (ARS-UCD1.2, provided by GenBank), accessed via Ensembl Release, employing Hisat2 (v2.1.0). The sorting of SAM files and the counting of transcript reads were facilitated by SAMtools, HTSeq-count, and RSEM. Analysis for a differentially expressed gene (DEG) was executed using DESeq2 (v1.10.1), applying a *p*-adjust (BH) ≤ 0.1 and a fold-change (FC) > 2 as criteria. Gene Ontology (GO) and Kyoto Encyclopedia of Genes and Genomes (KEGG) pathway analysis focused on DEG between SCK and control (CON) groups of cows.

### 2.6. Quantitative RT-PCR

Real-time quantitative reverse transcription PCR (qRT-PCR) was conducted to validate the critical candidate genes identified by adipose tissue transcriptomic analyses. The mRNA expression levels of adiponectin (ADIPOQ), adiponectin receptors 1 and 2 (ADIPOR1 and ADIPOR2), AKT serine/threonine kinase 2 (AKT2), ceramide synthase 2 and 5 (CERS2 and CERS5), fatty acid synthase (FASN), hormone-sensitive lipase (LIPE; also called HSL), protein kinase C zeta (PRKCZ) and serine palmitoyltransferase long chain base subunit 1 and 2 (SPTLC1 and SPTLC2) were determined by qRT-PCR. These genes were selected based on their role in the sphingolipid metabolism pathway and differential expression in RNA sequencing results. The design of primers was facilitated by Primer-BLAST software (version 2.15.0; available at http://www.ncbi.nlm.nih.gov/tools/primer-blast/, accessed on 30 November 2023), with the primer sequences detailed in Table S3. mRNA was isolated using the RNeasy Plus Mini Kit (Qiagen, Germantown, MD), and the synthesis of complementary DNA (cDNA) was carried out with the High-Capacity cDNA Reverse Transcription Kit (Thermo Fisher Scientific), adhering to the provided instructions. Quantitative PCR involved mixing 4 μL of diluted cDNA with 6 μL of a solution containing 5 μL of SYBR Green master mix (Accurate Biology, Hunan, China), 0.4 μL of each primer at 10 μM concentration, and 0.2 μL of DNase/RNase-free water. This procedure was executed on a Roche LightCycler 96 System (Basel, Switzerland), initiating at 95 °C for 4 min, followed by 40 cycles of 20 s at 95 °C, and 40 s at the primers’ annealing temperature with fluorescence measurement. A melting curve analysis, heating from 70 to 95 °C in 0.5 °C increments every 5 s with fluorescence recording, confirmed the specificity of amplification for each primer pair, with efficiencies between 95% and 105%. Samples were analyzed in triplicate, incorporating a 7-point standard curve and a control without a template. The 2^−ΔΔCt^ method was employed for data analysis. Expression levels of target genes were normalized to the geometric mean of housekeeping genes glyceraldehyde-3-phosphate dehydrogenase (GAPDH), β-actin (ACTB), and ribosomal protein S9 (RPS9), which were verified as stable in adipose tissue [26,27,28].

### 2.7. Serum and Adipose Tissue Untargeted Lipidome Analysis

Lipids were extracted from serum using a method outlined by Draijer et al. [29] and from adipose tissue following the procedure described by Chaves-Filho et al. [30]. The extracted lipids were then dissolved in a solvent mixture containing acetonitrile, isopropanol, chloroform, and water in a ratio of 35:35:20:10 (*v*/*v*/*v*/*v*). These samples underwent analysis via Orbitrap high-resolution tandem mass spectrometry (Q Exactive HF, Thermo Scientific, Waltham, MA, USA), coupled with Vanquish ultra-high performance liquid chromatography (UHPLC) (Thermo Scientific). An ACQUITY UPLC-HSS C18 column (2.1 mm × 150 mm × 2.5 μm; Waters, Milford, MA, USA) was used for lipid separation. Mobile phase A was a mix of acetonitrile and water (60:40, *v*/*v*, with 0.1% formic acid and 10 mmol/L ammonium formate), and mobile phase B was composed of isopropanol and acetonitrile (90:10, *v*/*v*, with 0.1% formic acid and 10 mmol/L ammonium formate). The flow rate was set at 0.4 mL/min, starting with 40% of solvent B, increasing to 43% at 2 min, 50% at 2.1 min, then to 54% at 12 min, and to 70% at 12.1 min. Solvent B was raised to 99% by 18 min, then dropped back to 40% at 18.1 min, followed by column re-equilibration for 4.9 min. A 2 μL sample volume was injected for analysis.

Lipid identification involved matching against an in-house lipidomics database and major public databases like the LIPID MAPS Structure Database (LMSD) and the Human Metabolome Database (HMDB), using retention time, precise mass, and fragmentation patterns. To illustrate differences in the lipidome between treatments, an orthogonal partial least squares discriminant analysis (OPLS-DA) scores plot was created. Lipid species were classified as significantly differentially abundant based on their variable importance in projection (VIP) scores from the OPLS-DA model, with selection criteria being VIP > 1 and *p*-value < 0.05.

### 2.8. Targeted Lipidome for Sphingolipids in Adipose Tissue

Sphingolipids were measured using LC-MS/MS, specifically with a QTRAP^®^ 6500+ system (AB Sciex, Framingham, MA, USA) connected to an ExionLC™ AD UPLC system (AB Sciex). Lipids were separated using a reversed-phase method on an ACQUITY HSS T3 column (2.1 mm × 100 mm × 1.8 μm), with a flow rate of 0.35 mL/min at 40 °C. Mobile phase A was a mix of acetonitrile and water (60:40, *v*/*v*, with 0.1% formic acid and 10 mmol/L ammonium formate), while mobile phase B was isopropanol and acetonitrile (90:10, *v*/*v*, with 0.1% formic acid and 10 mmol/L ammonium formate). The gradient schedule was set as follows: initial to 1.5 min at 37% B; then a gradual increase to 98% B at 20 min, holding for 2 min. The injection volume for samples was 5 μL. Electrospray ionization (ESI) source conditions included curtain gas at 20 psi, ion source gases 1 and 2 at 55 psi, a source temperature of 500 °C, an ion spray voltage of 5500 V, with entrance potentials set at 7 V and 12 V. Sphingolipids were detected using multiple reaction monitoring (MRM).

The UPLC–MS/MS data, acquired in MRM mode, were recorded using Sciex Analyst software (version 1.7.3) and analyzed with Sciex MultiQuant software (version 3.0.3; AB Sciex). Quantitative analysis employed an 8-point calibration curve with standards including Cer (d18:1/16:0), Cer (d18:1/17:0), Cer (d18:1/18:0), Cer (d18:1/24:0), Cer (d18:1/24:1), Cer (t18:0/24:0), Cer (d18:0/18:0)-OH, Cer (d18:1/18:0(2OH)), CerP (d18:1/8:0), SM (d18:1/17:0), SM (d18:1/18:0), SPH (d18:0), SPH (d18:1). Protein levels were determined using the Bradford assay (Thermo Fisher Scientific), and sphingolipid content was adjusted based on these protein concentrations.

### 2.9. Statistical Analysis

The data of 16 selected cows was used for statistical analysis. The DMI (0–30 d postpartum), milk production (0–30 d postpartum), BW (0–30 d postpartum), BCS (0–30 d postpartum), and serum BHBA (3 consecutive days for SCK diagnosis) were first checked for normality and analyzed using the PROC MIXED procedure of SAS version 9.4 (SAS Institute Inc., Madison, WI, USA). The model included time (week or day), group, and interaction of group and time as fixed effects. The time (week or day) was used as a repeated measure. First-order autoregressive (based on the lowest Akaike information criterion) was used for DMI, milk yield, BW, and BCS because their repeated measurements were equally spaced. 

The analyses of days in milk (DIM) at the start of blood sampling, DIM of the adipose tissue sampling, and other serum variables were conducted with one-way ANOVA using SAS 9.4 software (SAS Institute Inc.). Prior to analysis, the normality of all data was assessed using the UNIVARIATE function in SAS. Data that did not follow a normal distribution were transformed using the Box-Cox method. If data were not normally distributed after the transformations, interquartile range (IQR) was used to detect outliers. Data points falling below 1.5 times the IQR of the lower quartile or above 1.5 times the IQR of the upper quartile were identified as outliers and removed from the analysis. 

A mean difference between groups was deemed significant if *p* < 0.05 and a tendency was noted for 0.05 ≤ *p* < 0.10. The results are presented as least squares means ± the standard error of the mean.

## 3. Results

### 3.1. Serum Metabolic Status Parameters

Serum glucose/lipid metabolism and liver function indices are shown in Figure 1. The serum concentration of NEFA was greater for SCK than CON (*p* < 0.001; Figure 1b). As expected, we observed a higher serum glucose level (*p* = 0.002; Figure 1c) in CON cows. No significant differences (*p* > 0.05) were observed in serum insulin, TG, TC, LDL-C, or leptin, which were altered between the two groups (Figure 1d–h). The serum concentrations of adiponectin (*p* = 0.022; Figure 1i) were lower in SCK cows than in CON cows. The activity of ALT tended to be greater in SCK cows than in CON cows (*p* = 0.052; Figure 1l), but total protein (Figure 1j), albumin (Figure 1k), and AST (Figure 1m) were not different (*p* > 0.10). The CON group had a greater RQUICKI value than the SCK group (Figure S1).

**Figure 2:**
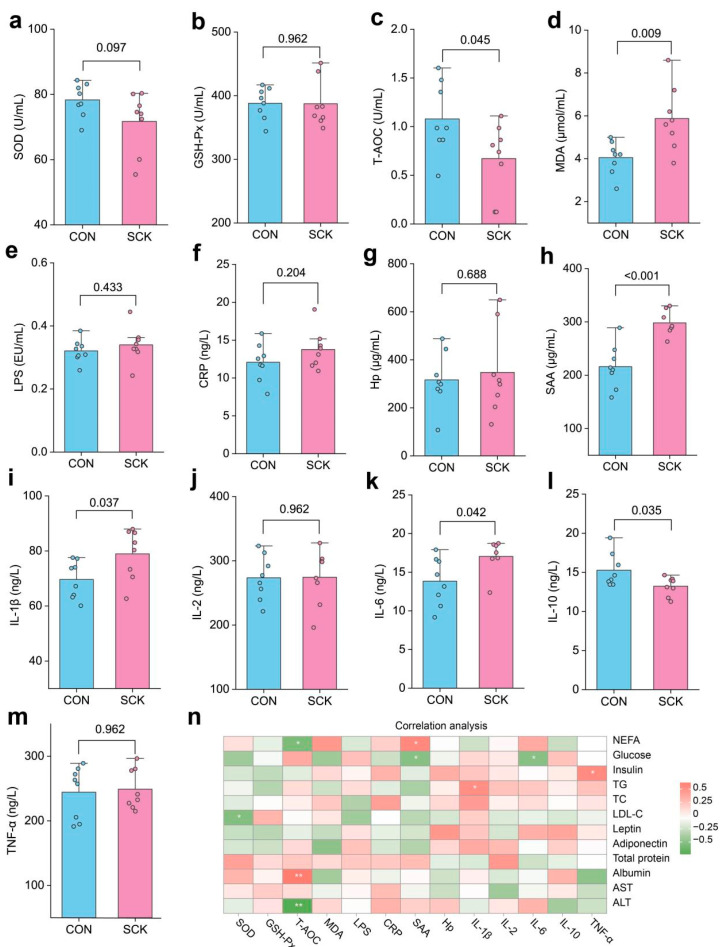
Serum oxidative status and inflammation indices. (**a**) SOD; (**b**) GSH-Px; (**c**) T-AOC; (**d**) MDA; (**e**) LPS; (**f**) CRP; (**g**) Hp; (**h**) SAA; (**i**) IL-1β; (**j**) IL-2; (**k**) IL-6; (**l**) IL-10; (**m**) TNF-α; (**n**) Pearson correlation analysis between serum glucose/lipid metabolism and liver function parameters and oxidative status and inflammation indices. * *p* < 0.05, ** *p* < 0.01. CON: healthy cows; SCK: subclinical ketosis; SOD: superoxide dismutase; GSH-Px: glutathione peroxidase; T-AOC: total antioxidant capacity; MDA: malonaldehyde; LPS: lipopolysaccharide; CRP: C-reactive protein; Hp: haptoglobin; SAA: serum amyloid A protein; IL: interleukin; TNF-α: tumor necrosis factor α; NEFA: nonesterified fatty acids; TG: triglyceride; TC: total cholesterol; LDL-C: low-density lipoprotein cholesterol; ALT: alanine aminotransferase; AST: aspartate aminotransferase.

Serum oxidative status and inflammation indices are depicted in Figure 2. SCK cows tended to have a lower activity of SOD (*p* = 0.097; Figure 2a). The activity of GSH-Px did not differ between the CON and SCK groups (*p* = 0.962; Figure 2b). The T-AOC was lower in the SCK group than in the CON group (*p* = 0.045; Figure 2c). SCK cows had a higher serum MDA than CON cows (*p* = 0.009; Figure 2d). The serum concentrations of LPS (Figure 2e), CRP (Figure 2f), Hp (Figure 2g), IL-2 (Figure 2j), and TNF-α (Figure 2m) were not different for CON and SCK cows (*p* > 0.05; Figure 2d). SCK cows had greater concentrations of SAA (*p* = 0.009; Figure 2h), IL-1β (*p* = 0.037; Figure 2i), and IL-6 (*p* = 0.042; Figure 2k). The serum concentration of IL-10 for SCK cows was lower than CON (*p* = 0.035; Figure 2l). We performed a Pearson correlation analysis between serum glucose/lipid metabolism, liver function parameters, and oxidative status and inflammation indices. Statistical significance (*p* < 0.05) and correlation coefficient (|r| > 0.5) indicate the correlations (Figure 2n). Serum NEFA was negatively correlated with T-AOC and positively correlated with SAA. Serum glucose was negatively correlated with IL-6 and SAA.

### 3.2. Biochemical Parameters of Subcutaneous Adipose Tissue

**Figure 3:**
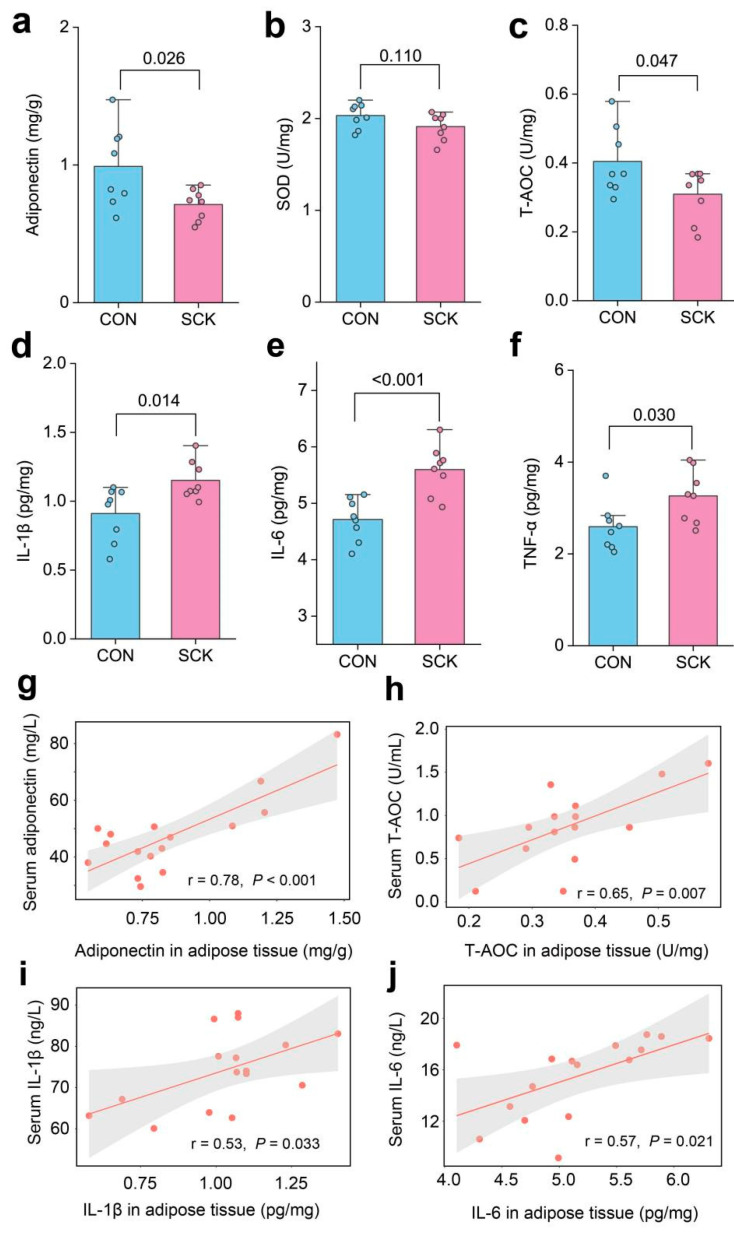
Biochemical parameters of subcutaneous adipose tissue. (**a**) Adiponectin; (**b**) SOD; (**c**) T-AOC; (**d**) IL-1β; (**e**) IL-6; (**f**) TNF-α; regression lines showing the associations between adipose tissue and serum concentrations of (**g**) adiponectin, (**h**) T-AOC, (**i**) IL-1β, and (**j**) IL-6. CON: healthy cows; SCK: subclinical ketosis; SOD: superoxide dismutase; T-AOC: total antioxidant capacity; IL: interleukin; TNF-α: tumor necrosis factor α.

The adipose tissue concentration of adiponectin (*p* = 0.026; Figure 3a) and activity of T-AOC (*p* = 0.047; Figure 3c) were lower in SCK cows than in CON cows. Compared with CON, SCK cows had greater concentrations of IL-1β (*p* = 0.014; Figure 3d), IL-6 (*p* < 0.001; Figure 3e), and TNF-α (*p* = 0.030; Figure 3f). The activity of SOD did not differ between the CON and SCK groups (*p* = 0.110; Figure 3b). We further assessed the association between serum and adipose tissue concentrations or activity of adiponectin, T-AOC, IL-1β, and IL-6, which were significantly changed parameters. Serum adiponectin, T-AOC, IL-1β, and IL-6 were positively correlated with their concentration or activity in adipose tissue (r > 0.50, *p* < 0.05; Figure 3g–j). 

### 3.3. Whole Blood and Adipose Tissue Transcriptome

Sixteen samples underwent sequencing, yielding 105.27 and 106.6 Gb of clean data following quality checks. Each sample achieved over 6.18 Gb and 6.01 Gb of clean data for whole blood and adipose tissue, respectively. The percentages of Q30 were 92.18–96.04%. Whole blood and subcutaneous adipose tissue from dairy cows expressed a total of 21,123 and 25,333 genes after alignment with the bovine genome, respectively. 

**Figure 4:**
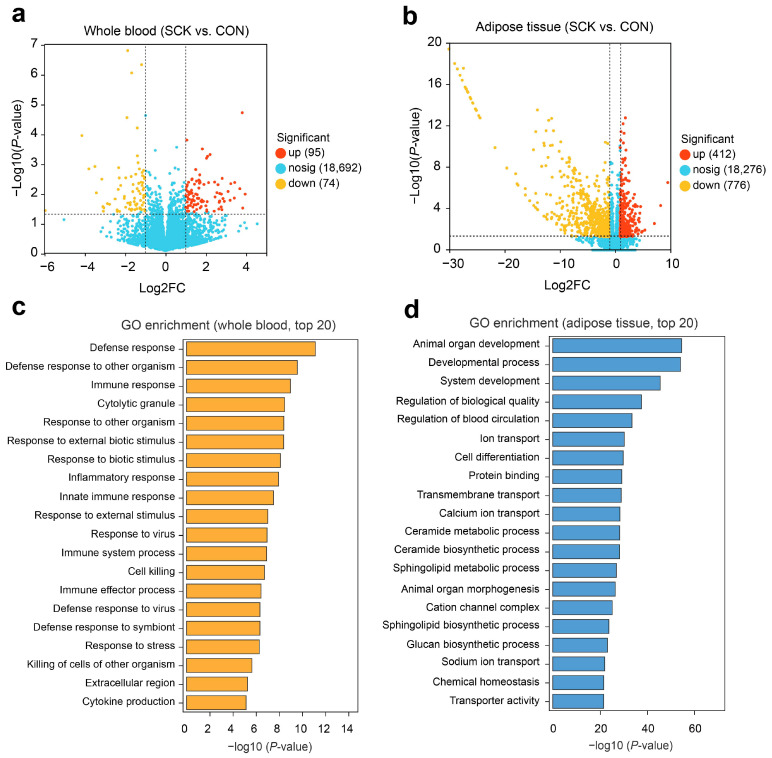
Transcriptomic profiling of whole blood and adipose tissue. Volcano plot showing the significant gene patterns of (**a**) whole blood and (**b**) adipose tissue. Up- and down-regulated DEG are represented by red and yellow dots, respectively. GO function enrichment analysis of DEG in (**c**) whole blood and (**d**) adipose tissue. CON: healthy cows; SCK: subclinical ketosis; GO: gene ontology.

Every DEG satisfied the selection thresholds of *p* (BH) < 0.05 and an absolute log2 fold change (|log2FC|) greater than 1. The distribution of DEG in the whole blood is presented in the volcano plot with 163 DEG, of which 95 were upregulated, and 74 were downregulated (Figure 4a). In the adipose tissue, 1188 DEG between the CON and SCK groups were identified. Of these, 412 genes were upregulated, and 776 were downregulated in the SCK group (Figure 4b). The corresponding gene lists (top 100 genes) for whole blood and adipose tissue are provided in Tables S4 and S5.

The GO enrichment analysis was carried out across three GO categories: biological processes (BP), cellular components (CC), and molecular functions (MF). In whole blood, significant GO terms were primarily linked to immune and inflammatory processes. These included defense response (GO:0006952), defense response to other organisms (GO:0098542), immune response (GO:0006955), cytolytic granule (GO:0044194), response to other organisms (GO:0051707), response to external biotic stimulus (GO:0043207), response to biotic stimulus (GO:0009607), inflammatory response (GO:0006954), and innate immune response (GO:0045087). For adipose tissue, 1188 DEG were enriched for multiple terms, such as animal organ development (GO:0048513), developmental process (GO:0032502), system development (GO:0048731), regulation of biological quality (GO:0065008), regulation of blood circulation (GO:1903522), ion transport (GO:0006811), and cell differentiation (GO:0030154), as shown in Figure 4d. Additionally, there was significant enrichment in genes involved in the ceramide metabolic process (GO:0006672), the ceramide biosynthetic process (GO:0046513), the sphingolipid metabolic process (GO:0006665), and the sphingolipid biosynthetic process (GO:0030148). 

The KEGG database was used for the enrichment analysis of biological pathways. Compared with the control, significant pathways in the whole blood were enriched in SCK cows, including influenza A (ko05164), NOD-like receptor signaling pathway (ko04621), apoptosis (ko04210), and Type I diabetes mellitus (ko04940) (Figure S2). Pathways related to the immune response and inflammation were also enriched, such as cytokine-cytokine receptor interaction (ko04060), IL-17 signaling pathway (ko04657), TGF-beta signaling pathway (ko04350), and TNF signaling pathway (ko04668). 

**Figure 5:**
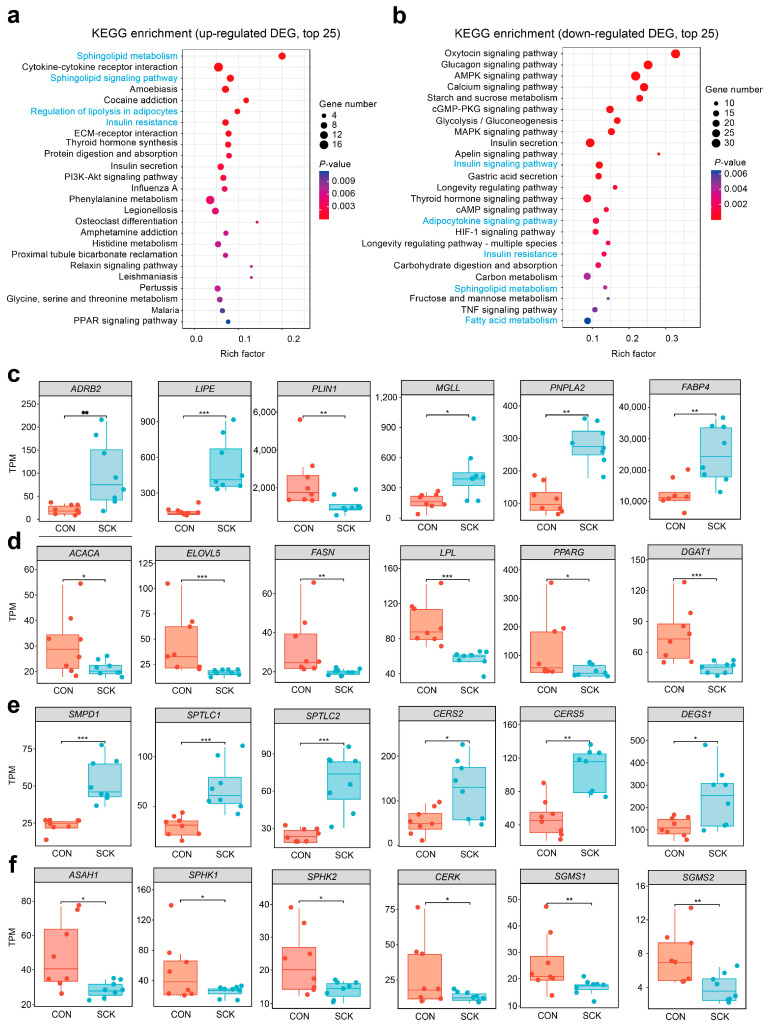
Representative differential genes and KEGG enrichment analysis in adipose tissue. (**a**) KEGG pathway enrichment analysis for upregulated DEG (top 25 terms); (**b**) KEGG pathway enrichment analysis for downregulated DEG (top 25 terms); transcripts per million (TPM) of selected gene representatives of (**c**) lipolysis, (**d**) lipogenesis (**e**) ceramide biosynthesis, and (**f**) ceramide catabolism. DESeq2 package (version 1.44.0) was used to identify the DEGs using a cutoff threshold of adj. *p* < 0.05 and fold change (FC) > 2. The asterisks indicate statistically significant differences and correspond to * *p* < 0.05, ** *p* < 0.01, and *** *p* < 0.001. CON: healthy cows; SCK: subclinical ketosis; KEGG: Kyoto Encyclopedia of Genes and Genomes.

We also identified enriched pathways using KEGG enrichment analysis on adipose tissue DEG. Increases in sphingolipid metabolism (ko00600), cytokine–cytokine receptor interaction (ko04060), sphingolipid signaling pathway (ko04071), regulation of lipolysis in adipocytes (ko04923), and insulin resistance (ko04931) was among the top 10 terms prioritized by KEGG analysis (Figure 5a). Decreased terms were related to the insulin signaling pathway (ko04910), the adipocytokine signaling pathway (ko04920), insulin resistance (ko04931), sphingolipid metabolism (ko00600), and fatty acid metabolism (ko01212). 

To better depict expression levels in individual samples and the variance of expression within the groups, transcripts per million (TPM) are displayed for representative genes. These genes are involved in lipolysis (e.g., *ADBR2*, *LIPE*, *PLIN1*, *MGLL*, *PNPLA2*, *FABP4*; Figure 5c), lipogenesis (e.g., *ACACA*, *ELOVL5*, *FASN*, *LPL*, *PRARG*, *DGAT1*; Figure 5d), ceramide biosynthesis (e.g., *SMPD1*, *SPTLC1*, *SPTLC2*, *CERS2*, *CERS5*, *DEGS1*; Figure 5e), ceramide catabolism (e.g., *ASAH1*, *SPHK1*, *SPHK2*, *CERK*, *SGMS1*, *SGMS2*; Figure 5f), insulin signaling pathway (e.g., *INSR*, *PPP2CA*, *PRKCZ*; Figure S3a), adiponectin production (e.g., *ADIPOQ*, *ADIPOR1*, *ADIPOR2*; Figure S3b), and inflammatory and oxidative status (e.g., *CCL2*, *CCL5*, *IL6*, *IL1B*, *IL18*, *TLR4*, *NFKB1*, *CASP1*, *GCLC*, *SOD1*, *SOD2*, *HIF1A*; Figure S3c). Genes related to lipolysis (*ADBR2*, *LIPE*, *MGLL*, *PNPLA2*, *FABP4*), ceramide biosynthesis (*SMPD1*, *SPTLC1*, *SPTLC2*, *CERS2*, *CERS5*, *DEGS1*), and inflammatory response (*CCL2*, *CCL5*, *IL6*, *IL1B*, *IL18*, *TLR4*, *NFKB1*, *CASP1*) were up-regulated, whereas genes related to lipogenesis (*ACACA*, *ELOVL5*, *FASN*, *LPL*, *PRARG*, *DGAT1*), ceramide catabolism (*ASAH1*, *SPHK1*, *SPHK2*, *CERK*, *SGMS1*, *SGMS2*), and adiponectin production (*ADIPOQ*, *ADIPOR1*, *ADIPOR2*) were down-regulated in SCK cows. SCK cows displayed lower adipose tissue expression of genes involved in insulin signaling pathway (*INSR*) antioxidant enzyme (*GCLC*, *SOD1*, *SOD2*), and had higher expression of genes related to insulin resistance (*PPP2CA* and *PRKCZ*) and oxidative stress (*HIF1A*).

To confirm the gene expression patterns identified in adipose tissue through RNA sequencing, we selected genes involved in lipolysis, lipogenesis, adiponectin production, ceramide metabolism, and insulin signaling for qRT-PCR testing. Results from qRT-PCR for these 11 differentially expressed genes matched the transcript per million (TPM) values obtained from RNA sequencing (Figure S4). This consistency between two distinct methodologies reinforces the reliability and precision of the RNA sequencing approach in reflecting gene expression alterations linked to metabolic disturbances in adipose tissue.

### 3.4. Serum and Adipose Tissue Lipidome

**Figure 6:**
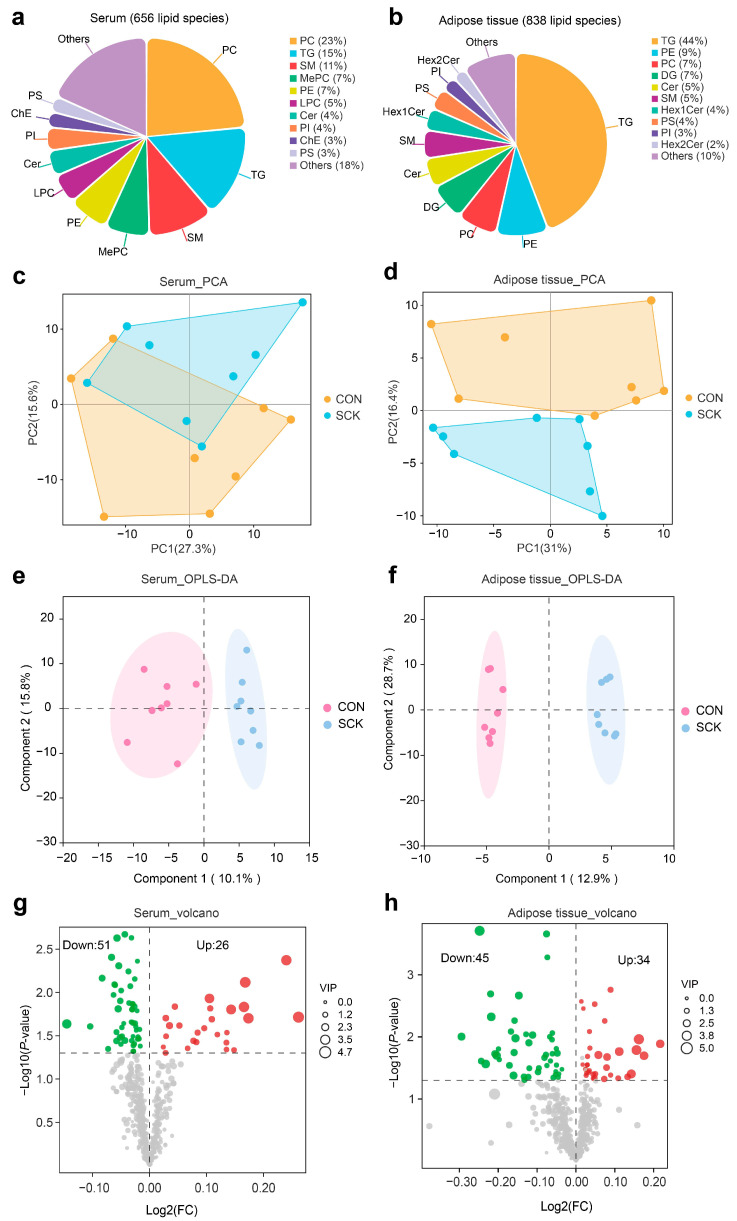
Serum and adipose tissue lipidome analysis. Identified lipid feature number and class in (**a**) serum and (**b**) adipose tissue samples. Scores plot of principal component analysis (PCA) of lipid species in (**c**) serum and (**d**) adipose tissue. Scores plot of the orthogonal partial least squares discriminant analysis (OPLS-DA) of lipid species in (**e**) serum and (**f**) adipose tissue. Volcano plots of differential lipid species in (**g**) serum and (**h**) adipose tissue. The red circles means up-regulated lipids; The green circles means down-regulated lipids.

A lipidomics analysis was used to further investigate lipid metabolism alterations under SCK to detect the total lipid classes in serum and adipose tissue. We identified 656 lipids (25 major lipid classes) in serum from 16 samples (Figure 6a). A total of 838 lipid species were identified in adipose tissue samples (Figure 6b). Phosphatidylcholine (PC, 23%) emerged as the most abundant lipid in serum samples, with TG (15%) and SM (11%) following closely. For adipose tissue, TG (44%) was the most abundant, followed by phosphatidylethanolamine (PE, 9%) and (PC, 7%). The unsupervised PCA was used to visualize the lipid differences between the groups. The PCA plots indicate that CON and SCK serum samples do not exhibit distinct separation (Figure 6c), while a significant divergence between the two groups in adipose tissue was observed (Figure 6d). The supervised OPLS-DA plots reveal that CON and SCK samples were well separated (Figure 6e,f). Qualitatively, the adipose tissue samples separated more than the serum samples, which suggests more variability in the adipose tissue.

**Figure 7:**
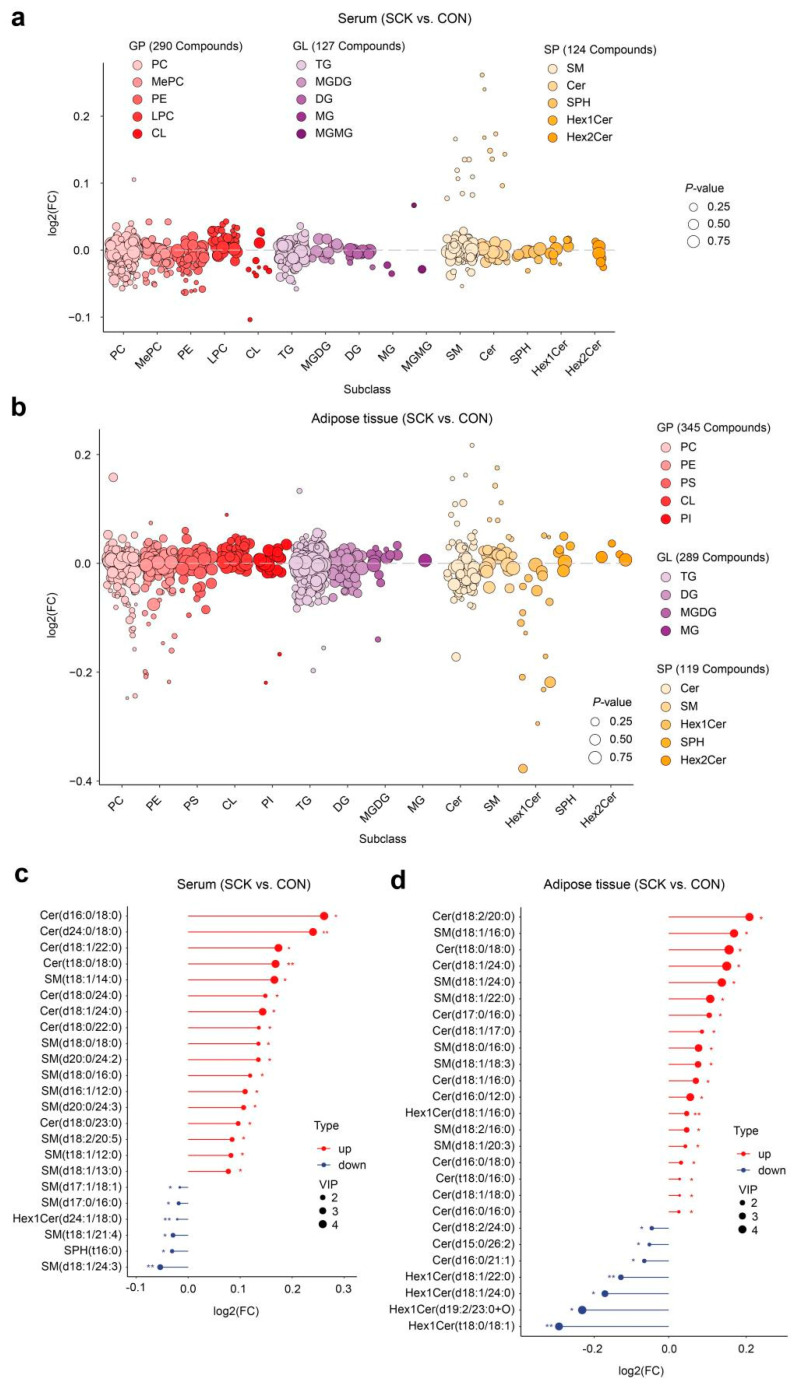
The expression of important lipid metabolites. Bubble plot of lipids in each main class and subclass of (**a**) serum and (**b**) adipose tissue samples. Analysis to identify differential altered sphingolipids in (**c**) serum and (**d**) adipose tissue. * *p* < 0.05 and ** *p* < 0.01.

In our study, the VIP value (VIP > 1) obtained from the OPLS-DA model along with the *p*-value (*p* < 0.05) were applied to identify differential lipid species observed in CON and SCK samples. There were 26 lipid species (e.g., Cer (d16:0/18:0), Cer (d24:0/18:0), Cer (d18:1/22:0), Cer (t18:0/18:0)) that were significantly up-regulated and 51 species (e.g., BisMePA (20:1/20:2), CL (18:3/18:0/18:2/20:0), PS (20:0e/22:4), PS (18:0e/22:4)) that were significantly down-regulated in the serum of SCK cows compared with CON (Figure 6g and Table S6). The volcano plot illustrates that 34 lipids increased (e.g., Cer (d18:2/20:0), SM (d18:1/16:0), Cer (t18:0/18:0), Cer (d18:1/24:0)) or 45 lipids decreased (e.g., Hex1Cer (t18:0/18:1), PC (10:1e/23:0), PC (18:0e/22:4), Hex1Cer (d19:2/23:0+O)) in SCK cows relative to CON cows (Figure 6h and Table S7). Lipid species belonging to SM and Cer were increased under SCK, and PE and PC dropped in response to SCK in serum or adipose (Figure 7a,b). The fold change in all significantly altered sphingolipid metabolites in serum and adipose tissue is clearly depicted in Figure 7c and Figure 7d, respectively. 

**Figure 8:**
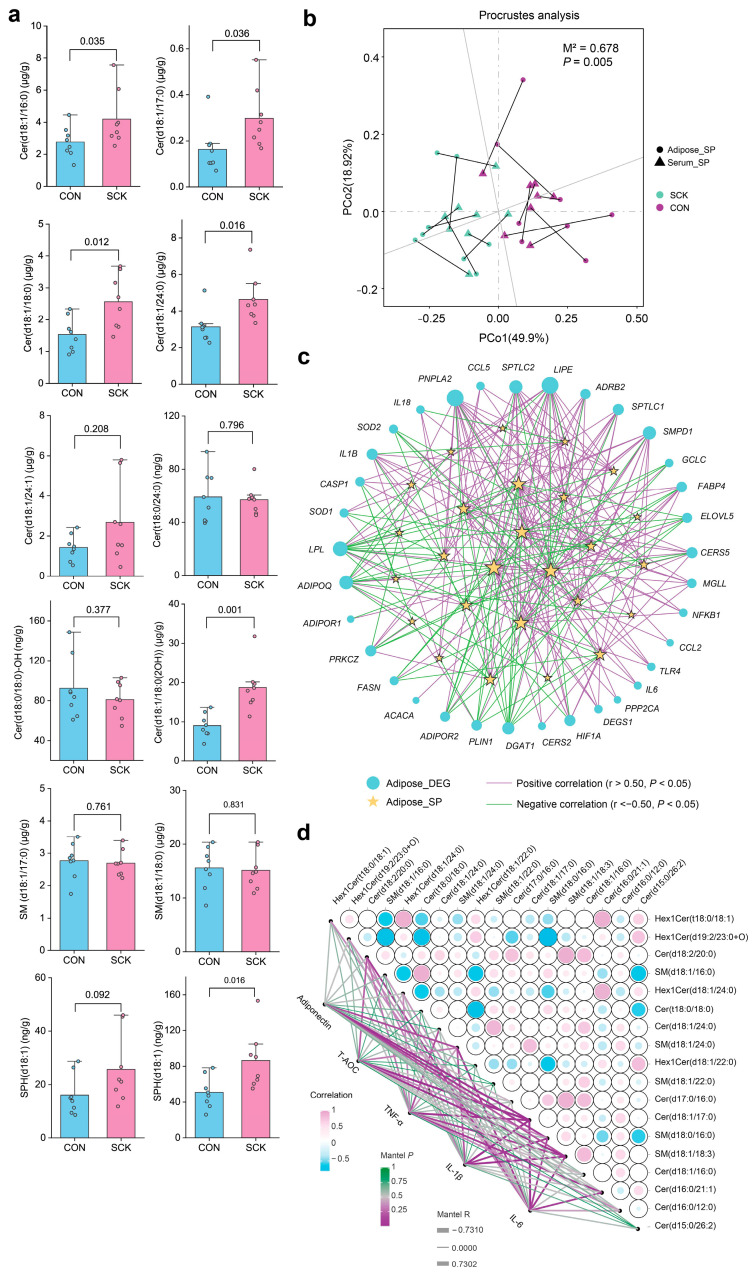
Targeted lipidomic analysis and association analysis of differential lipid species in adipose tissue. (**a**) Targeted lipidomic analysis of sphingolipids (SP) in adipose tissue. (**b**) The correlation between differential SP in serum and adipose tissue samples by Procrustes analysis. (**c**) Association network between differentially expressed genes and SP in adipose tissue based on Pearson correlation coefficients. (**d**) The heatmap on the right depicts the correlations between differential sphingolipid species in adipose tissue (top 18 ranked by |log2(FC)|). The network on the left depicts the correlations of adiponectin, T-AOC, TNF-α, IL-1β, and IL-6 with differential sphingolipid species in adipose tissue.

Targeted lipidomic analysis was performed to detect and determine the sphingolipid levels in adipose tissue samples. Among them, 12 sphingolipid features were identified using standards. Compared with CON, SCK cows had greater concentrations of Cer (d18:1/16:0), Cer (d18:1/17:0), Cer (d18:1/18:0), Cer (d18:1/24:0), Cer (d18:1/18:0(2OH)) and SPH (d18:1) in adipose tissue (Figure 8a). Targeted lipidome further verified the up-regulation of sphingolipid in SCK cows relative to CON.

We performed a Procrustes analysis within all samples from different groups to assess the overall associations between the lipidome and the transcriptome. The correlation is considered significant when *p* < 0.05. There was a consistent and significant inter-omics relationship between the differential lipid species and the DEG in adipose tissue across the different groups (Figure S5a; M2 = 0.73, *p* = 0.03), indicating that metabolic gene expression might alter the lipidomic profile in adipose tissue. However, relatively low correlations were observed in the relationships between DEG in whole blood and serum differential lipids (Figure S5b; M2 = 0.96, *p* = 0.75), DEG in adipose tissue and serum differential lipids (Figure S5c; M2 = 0.79, *p* = 0.07), and DEG in whole blood and adipose tissue (Figure S5c; M2 = 0.96, *p* = 0.78). Next, we investigated the correlations between differential sphingolipids in serum and adipose tissue. Serum differential sphingolipids showed a significant correlation with differential sphingolipids in adipose tissue (Figure S5c; M2 = 0.68, *p* = 0.005). 

We constructed an integrated omics network to examine relationships between lipids and genes by calculating the correlations between representative DEG and differential sphingolipids in adipose tissue. Statistical significance (*p* < 0.05) and the Pearson correlation coefficient (|r| > 0.5) indicate the correlations. Figure 8c shows genes relevant to lipolysis (e.g., *LIPE*, *ADRB2*, *MGLL*), ceramide biosynthesis (e.g., *SPTLC1*, *SPTLC2*, *DEGS1*), inflammation (e.g., *CCL5*, *IL1B*, *IL6*, *NFKB1*), and insulin resistance (e.g., *PPP2CA*, *PRKCZ*) were positively correlated with sphingolipid species. Genes related to lipolysis (e.g., *ELOVL5*, *LPL*, *DGAT1*), adiponectin (e.g., *ADIPOQ*, *ADIPOR*), and antioxidant enzymes (e.g., *SOD2*, *GCLC*) were negatively correlated with sphingolipid species. Differential sphingolipid species in adipose tissue were also significantly correlated with each other (Figure 8d; *p* < 0.05, |r| > 0.5). Adipose tissue concentrations of TNF-α, IL-6, and IL-1β were positively correlated with sphingolipid species (Figure 8d; *p* < 0.05, r > 0.5), such as Cer (d16:0/12:0), Cer (d16:0/21:1), Cer (d18:1/17:0), and SM (d18:0/16:0). Adipose tissue levels of adiponectin and T-AOC were negatively correlated with Cer(d17:0/16:0), Cer (d18:1/17:0), SM (d18:1/18:3), and SM (d18:1/24:0) (*p* < 0.05, r < −0.5). We further examined the associations between serum biochemical parameters and differential sphingolipid species in serum or adipose tissue using Pearson correlation. Serum concentrations of NEFA, MDA, SAA, and IL-6 were positively correlated with up-regulated sphingolipid species in serum and adipose tissue (*p* < 0.05, r > 0.5), such as SM (d20:0/24:2), SM (d20:0/24:3), Cer (d18:0/23:0), and SM (d18:1/16:0) (Figure S6). Serum levels of adiponectin, T-AOC, and IL-10 were negatively correlated with up-regulated sphingolipid species in serum and adipose tissue (*p* < 0.05, r < −0.5), such as SM (t18:1/14:0), Cer (d18:0/24:0), and Cer (d18:1/24:0). The RQUCKI value was also negatively correlated with up-regulated sphingolipid species in serum and adipose tissue (*p* < 0.05, r < −0.5). 

## 4. Discussion

The links among NEB, reproductive health, and metabolic disorders in dairy cows are well-documented [6,31]. Ketosis, a condition marked by the inefficient utilization of body fat during early lactation, leads to low blood glucose levels and high ketone body levels [32]. This condition triggers an increase in the breakdown of fats, releasing NEFA into the bloodstream when glucose levels drop [3]. Consistent with this understanding, our findings indicate higher levels of NEFA and reduced glucose levels in cows with SCK. The process of fat breakdown during the transition phase is driven by both conventional and inflammation-related pathways [33,34], starting with signals from β-adrenergic, growth hormone, and natriuretic peptide receptors for the conventional pathway. These activate adenylyl or guanylyl cyclases that increase the production of cAMP and cGMP, which are second messengers that turn on protein kinases A and G. Both kinases phosphorylate HSL, PLIN1, adipose triglyceride lipase, and its coactivator CGI-58, leading to triglyceride hydrolysis (i.e., lipolysis). Consistent with recent findings from Chirivi et al. [35] on adipose tissue in CK cows, our study also found an increase in the expression of lipase genes (such as *LIPE*, *MGLL*, *PNPLA2*) and a decrease in lipogenesis-related genes (including *ACACA*, *ELOVL5*, *FASN*, *PPARG*). This suggests that the root of lipolysis imbalance in these animals might be traced back to changes in gene expression. These results corroborate earlier descriptions of adipose tissue dysfunction in CK cows, as detailed by Chirivi et al. [35], who also noted a decrease in Akt phosphorylation, leading to a reduced inhibition of lipolysis when adipose tissue was exposed to insulin. In our study, the mRNA abundance of *AKT* and *INSR* (encoding insulin receptor) was decreased in SCK cows, indicating that insulin sensitivity might be impaired in adipose tissue. Because insulin is an antilipolytic hormone, insulin resistance in SCK cows can further increase adipose tissue lipolysis [36]. The factors contributing to insulin resistance in adipose tissue are complex and seem to stem from a mix of excessive lipid accumulation, inflammation, imbalances in adipokine levels, genetic predispositions, and mitochondrial dysfunction [37]. Our research highlighted a disturbance in sphingolipid homeostasis as a direct cause of reduced insulin sensitivity within adipose tissue.

**Figure 9:**
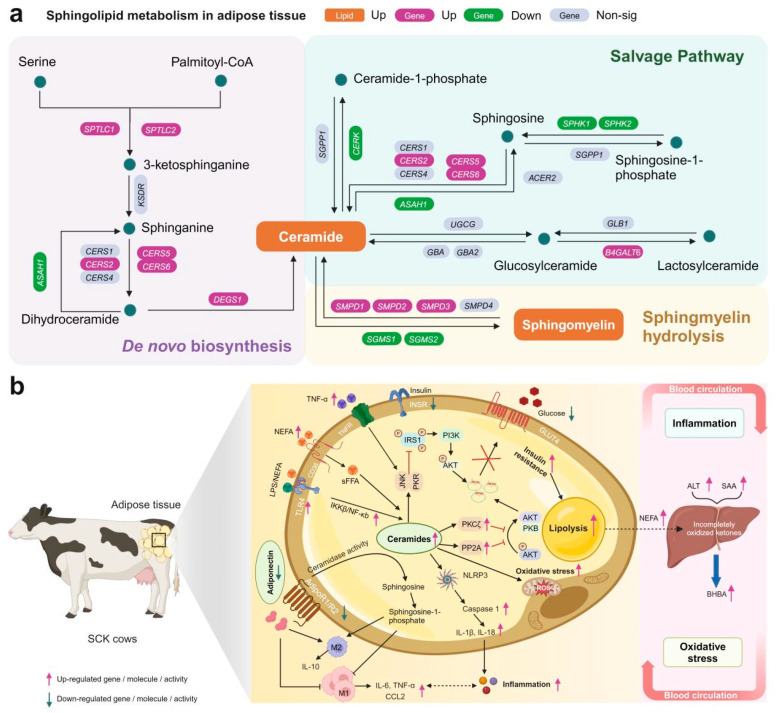
Summary of main findings on the ceramide metabolism in the present study. Integrated sphingolipid metabolism pathway analysis (**a**) and the ceramide-centric mechanisms of metabolic disorders in adipose tissue of SCK cows (**b**).

Increased NEFA availability can stimulate de novo ceramide synthesis in ruminants [38]. Our global lipidome data revealed increases in Cer and SM in serum and adipose tissue of SCK cows, compared with control. Ceramide concentrations are determined by the total produced through various enzymatic processes, including de novo synthesis, salvage, lysosomal degradation, and hydrolysis [39]. In this study, we observed that in cows with SCK, the expression of genes related to ceramide synthesis was upregulated. In contrast, the expression of genes involved in ceramide catabolism was downregulated (Figure 9a). These changes led to ceramide accumulation in adipose tissue, which may have contributed to the increased circulating levels of ceramide. Sphingolipids are crucial for their dual functions as critical structural elements of membranes and as molecules that promote or inhibit cell survival and apoptosis. Therefore, their concentrations must be meticulously regulated to maintain cellular integrity [40]. An imbalance in sphingolipid metabolism is associated with the development of insulin resistance and metabolic disorders linked to obesity [39,41]. We also found significant relationships between ceramide species and indicators of insulin sensitivity (*RQUICKI* and genes *INSR*, *PPP2CA*, *PRKCZ*). 

There is evidence causally linking ceramides to reduced systematic insulin sensitivity in transition cows. Research has shown a positive correlation between most circulating ceramides and monohexosylceramides (GlcCer) with NEFA and a negative correlation with insulin sensitivity in dairy cows [42,43]. Kenéz et al. [44] in their work on Holstein bulls found that a decline in protein kinase B (PKB) activation and insulin receptor presence in liver and adipose tissues was linked to increased ceramide levels in these tissues. In this study, cows with SCK exhibited significantly lower RQUICKI indices than control cows, echoing findings that ketotic cows have lower RQUICKI values compared to healthy cows [45,46]. Reviews by McFadden and Rico [47] and Zhao et al. [48] have highlighted ceramide’s growingly recognized influence in dairy cows, specifically its role in hampering insulin-driven glucose uptake in bovine adipocytes and potentially facilitating the insulin resistance and milk production boost induced by somatotropin [47,49,50].

Studies in non-ruminant models have shown that ceramides disrupt AKT function, leading to a decreased movement of glucose transporter-4 (GLUT4) to the cell surface in muscle and fat cells in response to insulin [51]. While the deactivation of AKT is a key step, the mechanism by which ceramide contributes to insulin resistance also involves activating phosphatase and tensin homolog (PTEN) and protein phosphatase 2A (PP2A), and drawing protein kinase C-ζ (PKCζ) into caveolin-rich areas of the cell membrane [51]. The significantly downregulated gene expressions of *PPP2CA* and *PRKCZ* confirmed the blocking action of ceramide on insulin signaling (Figure 9b). 

Adiponectin, predominantly produced by fat cells, is abundantly present in the bloodstream [52]. High levels of fat breakdown seen in cows with SCK could lead to reduced adiponectin production in fat cells. Research, including studies on 3T3-L1 cells, has demonstrated that adiponectin release declines when lipolysis is stimulated by β-adrenergic substances [53]. Consistent with findings from Akgul et al. [54] and Mann et al. [55], cows that exhibited hyperketonemia after calving had diminished adiponectin levels. Furthermore, Kabara et al. [56] found that adiponectin exposure notably lowered TNF-α levels in monocytes activated by LPS, thus significantly diminishing their inflammatory activity. A decrease in adiponectin in the plasma of SCK cows might lead to enhanced inflammatory responses by monocytes, evidenced by elevated levels of IL-1β, IL-6, and TNF-α in fat tissue. By inhibiting lipolysis and enhancing fatty acid oxidation, adiponectin also reduces insulin resistance and lowers TG concentrations in muscles and the liver [57]. Therefore, reduced adiponectin production in adipose tissue could be a factor in increasing insulin resistance.

Adiponectin activates ceramidase through its receptors, AdipoR1 and AdipoR2, enhancing the breakdown of ceramide and leading to the production of the antiapoptotic compound, sphingosine-1-phosphate (S1P) [58] (Figure 9b). In the study, we found that the mRNA levels of genes encoding adiponectin and its receptors in adipose tissue were downregulated, which might further diminish the conversion of ceramide to S1P, leading to ceramide accumulation. Moreover, the concentration of adiponectin was negatively correlated with ceramide, further supporting the possibility of an adiponectin–ceramide axis coupling in adipose tissue of dairy cows. 

TNF-α can elevate ceramide levels in plasma and adipose tissue by inducing the expression of ceramide production genes like SPT, SMPD1, and SMPD3 [59,60]. Furthermore, TNF-α enhances the production of ceramide, which activates nuclear factor kappa B (NF-κB) and triggers cell apoptosis [61]. In this study, stimulation of TLR4 (a receptor critical for innate immunity) by NEFA and LPS led to increased ceramide synthesis through the activation of ceramide-synthesizing enzymes, mediated by NF-κB as a key factor in TLR-4 driven ceramide generation (Figure 9b). The mRNA abundances of NLR family pyrin domain containing 3 (*NLRP3*), caspase-1 (*CASP1*), *IL1B*, and *IL18* were greater in the blood of cows with SCK, and mRNA levels of *NFKB1*, *CASP1*, *IL1B*, and *IL18* and *IL-1β* concentration were also greater in adipose tissue of SCK cows. The findings show that the development of ketosis triggers the NF-κB signaling pathway and the NLRP3 inflammasome, leading to inflammation. In non-ruminant species, there is a well-documented association between the NLRP3 inflammasome and ceramide metabolism [62,63]. The NLRP3 inflammasome responds to lipotoxicity-induced rises in ceramide levels, promoting the activation of caspase-1 in macrophages and fat tissue [64]. Our data imply that disruptions in ceramide metabolism may significantly contribute to the onset of SCK by exacerbating inflammatory responses via the NLRP3 pathway.

Lipolysis partly induces adipose tissue macrophage (ATM) infiltration by excessive release of NEFA from adipocytes [65]. Uncontrolled lipolysis in SCK cows could be associated with ATM infiltration. A higher number of subcutaneous and visceral adipose tissue macrophages was observed in cows with high lipolysis [66]. Macrophages in adipose tissue are essential for both tissue repair (M2 macrophages) as well as mounting an inflammatory response (M1 macrophages) with the liberation of pro-inflammatory cytokines (e.g., TNF-α and IL-6), triggering the acute phase response. In our research, the genes associated with the M1 macrophage inflammatory response, such as *CCL2*, *IL6*, and *TNF-α*, were significantly elevated in the adipose tissue of cows with SCK compared to healthy cows. The heightened expression of CCL2 (macrophage chemotactic protein-1) and CCL5 (macrophage-derived chemoattractant) indicates that macrophages are drawn to adipose tissue, contributing to inflammatory responses [27]. In cows with SCK, the increased presence of macrophages in adipose tissue promotes lipolysis, thus perpetuating a cycle that links enhanced lipolysis with macrophage infiltration and inflammation.

In addition to energy buffering, the adipose tissue is an endocrine organ that modulates systemic metabolism, immune function, and inflammatory processes. This function is exerted through the transcription and translation of a plethora of proteins, termed adipokines (e.g., adiponectin), and lipid mediators of inflammation (e.g., oxylipins, sphingolipids) [67]. There is a significant correlation between sphingolipids in adipose tissue and serum sphingolipids. Therefore, changes in adipose tissue sphingolipids may affect systemic immune function and inflammation, and be reflected in the blood transcriptome and lipidome. GO analyses of whole blood DEG showed enrichment of pathways involved in immune function, including the defense response, immune response, inflammatory response, innate immune response, and immune system process, suggesting a response to endogenous stimuli. Specifically, several pattern recognition receptors and downstream inflammation-related signaling pathways, such as the NOD-like receptor signaling pathway, the IL-17 signaling pathway, the TGF-β signaling pathway, the TNF signaling pathway, and the NF-κB signaling pathway, were enriched. These results might indicate that increased circulating ceramide concentrations altered various immune processes in the blood cell. 

Increased serum biomarkers of inflammation (e.g., IL-1β, IL-6, IL-8, TNF-α, SAA, Hp) appear to be closely associated with ketosis in transition dairy cows [68,69]. In the present study, cows with SCK had higher levels of serum SAA, IL-1β, IL-6, IL-8, and TNF-α. Previous studies demonstrated that cows with ketosis had increased positive acute phase proteins (APP; i.e., lipopolysaccharide-binding protein, Hp, SAA) [68,70]. The observed rise in positive APP in this study indicates systemic inflammation in cows with SCK. Serum Hp and SAA are primarily synthesized by liver cells in reaction to inflammatory cytokines such as IL-6 and TNF-α, as well as glucocorticoids [71], a pattern also noted in our findings. The increase in IL-1β and IL-6 levels in SCK cows led to elevated SAA concentration. These systemic inflammatory signals can suppress appetite, induce liver damage, and escalate energy use by the immune system, thereby exacerbating the NEB in dairy cows [72]. IL-10, produced by regulatory lymphocytes, plays a protective role by inhibiting the activity of effector cells involved in harmful autoimmune responses [73]. A decrease in serum IL-10 in cows with SCK could be a factor in the heightened inflammatory response observed in these animals.

Oxidative stress arises from a disproportion between reactive oxygen species (ROS) and the body’s antioxidant defenses. Cows with ketosis are in a state of high-intensity energy metabolism, which leads to oxidation and antioxidant imbalance, resulting in oxidative stress and injury [69]. The adipose tissue activates antioxidant defenses, such as the GSH, SOD, and Nrf2 systems, and mitochondrial uncoupling, to neutralize free radicals that may be increased during the transition period. Evidence exists of oxidative stress in transition cows based on changes in plasma MDA and T-AOC [74,75]. Our results showed that the serum oxidation product MDA was significantly increased in SCK cows compared with the healthy cows. In addition, the T-AOC level was significantly reduced in SCK cows’ blood and adipose tissue. Our transcriptomics analysis revealed decreased mRNA levels of *CAT*, *SOD1*, and *SOD2* in blood and *GCLC*, *SOD1*, and *SOD2* in adipose tissue of SCK versus CON. These results suggest that dairy cows with SCK exhibit oxidative stress, which agrees with previous studies [76,77]. Mitochondria have been pinpointed as a key site affected by ceramide, which induces ROS production by interacting with complex III of the electron transport chain [78]. Moreover, an increase in ceramide levels within cells boosts the creation of ganglioside GD3 in the endoplasmic reticulum and facilitates its transfer to mitochondria [79]. Within mitochondria, ganglioside GD3 triggers the release of superoxide (O2•) at complex III [80], leading to oxidative stress in adipose tissue.

In SCK cows, blood flow and oxygen supply are both diminished due to the increased intercapillary distance caused by the rapid enlargement of adipose tissue and adipocyte sizes. In response to local hypoxia, adipose tissue produces hypoxia-inducible-factor-1α (HIF-1α), a transcription factor that in turn induces angiogenic growth factors [81]. Additionally, increased levels of HIF-1α have been linked to inflammation [82] and apoptosis within adipose tissue [83]. The stimulation of HIF-1α in adipocytes is known to enhance ceramide synthesis through SMPD3 activation [84]. Accordingly, we also found that the mRNA level of the gene encoding HIF-1α (*HIF1A*) was positively correlated with ceramide species.

Liver injury is the consequence of adipose tissue mobilization induced by the NEB in postpartum dairy cows. The use of liver-specific enzymes as indicators of liver function is a common method for assessing liver health. When liver cells sustain damage, ALT and AST are released into the bloodstream, increasing their activity levels in the blood. Our study observed that ALT levels tended to be higher in cows with SCK compared to CON, suggesting possible liver damage in ketotic cows. These observations align with previous research that has also found elevated levels of AST or ALT in the blood of dairy cows affected by ketosis [76,85,86].

A limitation of our study is that we characterized the profiling of transcriptome and lipidome in subcutaneous adipose tissue only and that levels of sphingolipids might differ from visceral adipose tissue, potentially underestimating an effect of disrupted sphingolipid metabolism on adipose tissue dysfunction. Retroperitoneal adipose tissue (RPAT) and SCAT in dairy cows have distinct characteristics in terms of insulin signaling, proinflammatory responses, and lipolytic activity [87,88,89]. Studies have shown that RPAT exhibits a higher sensitivity to the insulin signaling pathway, demonstrated by elevated phosphorylation of Akt and adenosine monophosphate-activated protein kinase (AMPK), along with increased expression of fatty acid synthase [87]. Furthermore, RPAT shows a higher expression of proinflammatory cytokines and chemokines at the mRNA level under conditions of energy surplus [88]. Leung et al. [90] observed variations in sphingolipid compositions between bovine RPAT and SCAT, notably finding higher ceramide levels in RPAT than in SCAT. Sphingolipids of the de novo synthesis pathway, such as sphinganine, dihydroceramide, and Cer, were more concentrated in RPAT than in SCAT [90]. Sphingolipids of the salvage pathway and the sphingomyelinase pathway, such as SPH, S1P, ceramide-1-phosphate, glycosphingolipid, and SM, were more concentrated in SCAT [90]. This unique distribution of sphingolipids may contribute to differences in insulin sensitivity, inflammation, and oxidative stress between RPAT and SCAT in cows with SCK. Further studies are needed to delve into the metabolic stimuli and pathways involving sphingolipids like Cer, SM, and SPH within adipose tissues to fully understand their role in the pathogenesis of SCK. 

## 5. Conclusions

Dairy cows with SCK are characterized by excessive lipolysis of adipose tissue, systemic inflammation, oxidative stress, and liver damage in dairy cows. Differentially expressed genes in adipose tissue associated with SCK were related to excessive lipolysis, sphingolipid metabolism, insulin resistance, oxidative stress, and inflammation. Elevated levels of fatty acids, inflammatory mediators, and decreased expression of adiponectin receptors are key contributors to the buildup of ceramides in both adipose tissue and circulation. These ceramides exacerbate insulin resistance and mitochondrial impairment, intensifying the inflammatory condition. This condition may indicate a self-reinforcing cycle of immunometabolic disorders and oxidative stress in SCK cows. Thus, adipose tissue may play an important role in connecting ceramide metabolism, insulin resistance, oxidative stress, and inflammation in SCK development. These results indicate that nutritional manipulation and pharmacological therapy aimed at reducing the levels of ceramide and downstream events should be a promising strategy for preventing ketosis. 

## Data Availability

The generated FASTQ files were uploaded to the Sequence Read Archive Database at the National Center for Biotechnology Information (PRJNA1076465 and PRJNA1074691). The datasets used and/or analyzed during the current study are available from the corresponding author upon reasonable request.

## Supplementary Materials

The following supporting information can be downloaded at: https://www.mdpi.com/article/10.3390/antiox13050614/s1, Table S1: Basic description of healthy cows (CON) and subclinical ketosis (SCK) cows; Table S2: Ingredients and chemical composition of the diet; Table S3: Bovine primers used in conventional real-time PCR analysis; Table S4: Differentially expressed genes (top 100 of expression level (TPM)) in the whole blood between CON and SCK cows; Table S5: Differentially expressed genes (top 100 of expression level (TPM)) in the adipose tissue between CON and SCK cows; Table S6: Differential lipid species in the serum samples between CON and SCK cows; Table S7: Differential lipid species in the adipose tissue samples between CON and SCK cows; Figure S1: Revised quantitative insulin sensitivity check index (RQUICKI) of health cows and subclinical ketosis cows; Figure S2: KEGG pathway enrichment analysis of whole blood (top 25 terms); Figure S3: Representative differential genes in adipose tissue; Figure S4: Validation of adipose tissue differential gene expression of RNA-seq by qPCR; Figure S5: Procrustes analysis of the relationship between differential lipid features and differentially expressed gene (DEG); Figure S6: Heatmap of association between serum biochemical parameter and differential sphingolipids in serum and adipose tissue.
