# Supplementary material for: Multi-Omics Reveals Disrupted Immunometabolic Homeostasis and Oxidative Stress in Adipose Tissue of Dairy Cows with Subclinical Ketosis: A Sphingolipid-Centric Perspective
Source: Antioxidants (Basel). 2024 May 17;13(5):614. doi: 10.3390/antiox13050614 (PMC11118941; doi:10.3390/antiox13050614)
Supplement: Supplementary file 1 [file antioxidants-13-00614-s001.zip › antioxidants-2977757-supplementary.pdf]

antioxidants

MDPI

Supplementary Materials

Table S1. Basic description of selected healthy cows (CON) and subclinical ketosis (SCK) cows[1]

Item[1]	Group		SEM	*P*-value		
	CON (n = 8)	SCK (n = 8)		Group	Time	Group×Time
Postpartum BW (kg)[2]	634	651	4.2	0.087	0.018	0.527
Postpartum BCS[2]	3.36	3.48	0.061	0.032	0.067	0.750
Postpartum DMI (kg)[3]	17.7	16.2	0.04	0.064	<0.001	0.469
Milk yield (kg)[3]	32.7	30.4	0.68	0.019	<0.001	0.673
Serum BHBA (mmol/L)[4]	0.33	2.06	0.39	<0.001	0.402	0.385
DIM at the start of blood sampling (d)	6.7	7.6	0.41	0.412	—	—
DIM of the adipose tissue sampling (d)	9.7	10.6	0.43	0.412	—	—

[1]BW = bodyweight; BCS = body condition score; DMI = dry matter intake; BHBA = β-hydroxybutyric acid; DIM = days in milk. [2]BW and BCS were recorded weekly from calving to 30 d postpartum. [3]Milk yield and DMI were recorded daily from calving to 30 d postpartum. [4]Blood sampled by puncture of coccygeal vessels from cows after milk ketone bodies detection, and the serum BHBA on three consecutive days were used for statistical analysis.

Table S2. Ingredients and chemical composition of the diet.

Item[1]	Prepartum	Early lactation
Ingredient, % of DM		
Corn silage	25.7	30.4
Alfalfa silage	-	7.84
Oat Hay	45.6	-
Alfalfa hay	-	10.4
Soybean meal	7.68	16.2
Soybean hull	7.74	3.65
Crushed corn	3.88	9.46
Corn steam flakes	-	9.30
Whole cottonseed	-	5.36
Corn gluten meal	4.08	-
Sugarcane molasses	-	2.59
DCAD supplement	1.01	1.01
Mineral and vitamin mix[2]	4.31	3.70
Chemical composition, % of DM		
DM, as fed %	51.2	47.8
CP	15.6	17.5
NFC[3]	33.2	38.5
NDF	43.5	33.2
ADF	23.5	19.3
EE	3.48	5.10
Ash	5.10	5.68
NE_L (Mcal/kg of DM)	1.38	1.69
DCAD[4]	−98.1	291

[1]DM = dry matter; DCAD = dietary cation-anion difference; CP = crude protein; NFC = non-fiber carbohydrates; NDF = neutral detergent fiber; ADF = acid detergent fiber; EE = ether extract; NE_L = net energy for lactation. [2]Prepartum premix: 1 kg of premix included 1,400,000 IU Vitamin A, 437,500 IU Vitamin D, 19,000 IU Vitamin E, 2,000 mg Cu, 3,600 mg Mn, 9,000 mg Zn, 120 mg Se, 160 mg I, 100 mg Co. Early lactation premix: 1 kg of premix included 150,000 IU Vitamin A, 35,000 IU Vitamin D, 2,000 IU Vitamin E, 250 mg Cu, 500 mg Mn, 1,000 mg Zn, 20 mg Se, 40 mg I, 25 mg Co. [3]NFC = DM − (ash + CP + EE + NDF). [4]DCAD (mEq/kg of DM) = (Na + K) − (Cl + S), where the unit of Na, K, Cl, and S is mEq/kg of DM.

Table S3. Bovine primers used in conventional real-time PCR analysis

Gene[1]		Sequence[2]	Accession No.
ADIPOQ	F	TGTTCCTCTTAATCCTGCCCA	NM_174742.2
	R	CCAACCTGCACAAGTTCCCTT	
ADIPOR1	F	AATGGGGCTCCTTCTGGTAAC	NM_001034055.1
	R	GCAGACCTTATACACGAACTCC	
ADIPOR2	F	GGAGTGTTCGTGGGCTTAGG	NM_001040499.2
	R	GCAGCTCCGGTGATATAGAGG	
AKT2	F	ACGTGGTGAATACATCAAGACC	NM_001206146.2
	R	GCTACAGAGAAATTGTTCAGGGG	
CERS2	F	ATGCTCCAGACCTTGTATGACT	NM_001034667.1
	R	CTGAGGCTTTGGCATAGACAC	
CERS5	F	CCACCCTTTTTGTGATCTTTGGC	NM_001102132.2
	R	CCTGGGTCTAATCCCTCCTGG	
FASN	F	AGAGATCCCGAGACGCTTCT	NM_001012669.1
	R	GCTTGGTCCTTTGAAGTCGAAGA	
LIPE	F	GATTTACGCACGATGACACAGT	NM_001040502.2
	R	ACCTGCAAAGACATTAGACAGC	
PRKCZ	F	CCACGAAGCAGCCATCCATCC	NM_001077833.2
	R	TGATTCAGCGGAGACAGGAGGAG	
SPTLC1	F	ACGAGGCTCCAGCATACCAT	NM_001034749.1
	R	TCAGAACGCTCCTGCAACTTG	
SPTLC2	F	GTGAGGAACGGGTACTTGAGG	NM_001099081.2
	R	CAACCAGCATGGGTGTTTCTT	
ACTB	F	GCCCTGAGGCTCTCTTCCA	NM_173979.3
	R	GCGGATGTCGACGTCACA	
GAPDH	F	TTCAACGGCACAGTCAAGG	NM_001034034.2
	R	ACATACTCAGCACCAGCATCAC	
RPS9	F	TTCCAGAGCGTTGGCTTAGG	NM_001101152.2
	R	CTCATCCAGCACCCCGATAC	

[1]Primers were designed to measure the abundance of the following transcripts: adiponectin (*ADIPOQ*), adiponectin receptor 1 and 2 (*ADIPOR1* and *ADIPOR2*), AKT serine/threonine kinase 2 (*AKT2*), ceramide synthase 2 and 5 (*CERS2* and *CERS5*), fatty acid synthase (*FASN*), hormone-sensitive lipase (*LIPE*; also called *HSL*), protein kinase C zeta (*PRKCZ*) and serine palmitoyltransferase long chain base subunit 1 and 2 (*SPTLC1* and *SPTLC2*).

[2]Primer sequences are given in a 5′ to 3′ orientation.

Table S4. Differentially expressed genes (top 100 of expression level (TPM)) in the whole blood between CON and SCK cows.

Gene name	Log2FC(SCK/CON)	*P*-adjust	Regulate
HBB	2.694	0.012	up
HBA	2.856	0.018	up
CASP1	1.157	0.047	up
ISG15	-2.287	0.004	down
GNLY	1.318	0.012	up
MX1	-1.123	0.005	down
TLR8	2.921	0.022	up
CTSW	1.256	0.011	up
TLR7	1.704	0.003	up
IFITM3	-1.322	0.010	down
OAS1Y	-2.317	0.002	down
ALAS2	2.553	0.011	up
MX2	-1.968	0.004	down
REEP1	2.665	0.024	up
PRF1	1.089	0.010	up
APOBEC3Z1	-1.156	0.007	down
GZMB	1.432	0.021	up
CERS2	1.612	0.027	up
NOD1	1.661	0.004	up
5S_rRNA	3.834	0.038	up
SPTLC1	1.766	0.022	up
PPP1R1B	-1.266	0.037	down
DHX58	-1.108	0.009	down
OAS1Z	-1.234	0.021	down
IL18	1.912	0.006	up
DEFB4A	-1.035	0.042	down
UBA7	-1.134	0.003	down
HBM	2.773	0.013	up
DDX58	-1.153	0.002	down
IL1B	1.505	0.013	up
ADGRG1	1.150	0.005	up
SMAD1	-1.088	0.017	down
MYO1A	-1.182	0.000	down
DLL1	-2.217	0.007	down
WEE2	1.237	0.031	up
BEX5	-1.146	0.030	down
GZMM	1.699	0.005	up
CAT	-1.662	0.041	down
IL11RA	1.063	0.007	up
SOD1	-1.745	0.029	down

TMIGD2	1.833	0.000	up
USF1	-1.249	0.012	down
CERK	-1.673	0.000	down
PTX3	-1.516	0.043	down
CPT2	1.138	0.031	up
TBXA2R	-1.546	0.023	down
FAM3B	-1.390	0.001	down
MYBPC2	1.084	0.006	up
MT1E	1.097	0.037	up
WNT9A	-1.866	0.025	down
FSD1	-1.617	0.011	down
TUBA1C	-1.400	0.008	down
IL3RA	-1.356	0.001	down
PTRHD1	1.442	0.006	up
RYR1	-1.395	0.000	down
FOXS1	-1.035	0.003	down
PLEKHF1	1.169	0.011	up
P2RX5	-1.212	0.040	down
ATP6V0A4	1.029	0.009	up
SMIM6	1.890	0.022	up
H2AC7	-1.016	0.048	down
CFB	-1.469	0.003	down
CCDC191	-1.014	0.014	down
CHRND	1.394	0.015	up
NXPH3	1.082	0.048	up
SPTAN1	1.135	0.019	up
SYT8	1.147	0.048	up
KLF1	1.323	0.020	up
MSMB	2.608	0.006	up
COL4A2	-1.869	0.000	down
FAIM2	1.157	0.013	up
AREG	-1.289	0.002	down
CXCL2	-1.075	0.014	down
ICAM4	2.647	0.040	up
FCGR1A	-1.695	0.003	down
CELA1	1.309	0.004	up
CP	1.362	0.035	up
ME3	1.381	0.021	up
MUC13	-1.081	0.002	down
KANK2	1.216	0.012	up
CAPN5	-3.795	0.002	down
IFNG	-2.336	0.009	down
AMPH	1.065	0.000	up
DOC2A	-1.039	0.004	down

PRKN	1.056	0.036	up
SOD2	-1.904	0.000	down
SLC39A8	-1.204	0.005	down
LTBP4	-1.784	0.041	down
TUBA3E	2.766	0.034	up
CSTB	-2.040	0.018	down
EFHD1	1.282	0.025	up
PPM1L	1.359	0.019	up
CD5L	2.049	0.048	up
SGCE	3.418	0.017	up
SP6	3.692	0.008	up
IGFBP7	1.238	0.012	up
RNF39	3.812	0.000	up
LYZ2	2.039	0.001	up
MYL3	1.528	0.029	up
CRYM	-1.601	0.006	down

FC: fold change; CON: healthy cows; SCK: subclinical ketosis.

antioxidants

MDPI

Table S5. Differentially expressed genes (top 100 of expression level (TPM)) in the adipose tissue between CON and SCK cows.

Gene name	Log2FC(SCK/CON)	*P*-adjust	Regulate
MYL2	-19.522	<0.001	down
COL1A1	1.694	0.047	up
DES	-4.365	<0.001	down
TPM2	-4.801	<0.001	down
COL1A2	1.698	0.041	up
COL3A1	2.005	0.013	up
ACTA1	-12.813	<0.001	down
TNNC1	-17.494	<0.001	down
FHL1	-1.438	0.048	down
MUSTN1	-2.104	0.003	down
TNNC2	-13.209	<0.001	down
ALDOA	-1.751	0.013	down
MYLPF	-14.285	<0.001	down
CRYAB	-4.271	<0.001	down
CA3	-16.177	<0.001	down
TNNT1	-12.448	<0.001	down
MYL12A	-1.170	0.008	down
TPM1	-1.507	0.003	down
CKM	-14.275	<0.001	down
MB	-12.727	<0.001	down
CSRP3	-17.664	<0.001	down
TPM3	-1.840	0.002	down
MYL6B	-11.623	<0.001	down
YBX3	-3.093	0.002	down
PDLIM3	-3.963	<0.001	down
TCAP	-5.874	<0.001	down
TNNI2	-11.278	<0.001	down
TNNI1	-3<0.001	<0.001	down
TNNT3	-8.498	<0.001	down
ATF4	-1.481	0.005	down
KLHL41	-11.941	<0.001	down
MYH7	-13.986	<0.001	down
EEF1A2	-6.475	0.018	down
PLIN4	1.315	<0.001	up
JUN	-1.810	<0.001	down
FOS	-3.153	<0.001	down
MYH2	-17.920	<0.001	down
IGFBP6	1.137	0.020	up
ACTN2	-14.694	<0.001	down
MYOZ1	-9.755	<0.001	down

MFAP5	1.183	0.016	up
DUSP1	-1.674	<0.001	down
CCNG1	-1.263	0.006	down
MYOT	-3<0.001	<0.001	down
NRAP	-11.411	<0.001	down
ATP2A1	-10.037	<0.001	down
LGALS3	1.054	0.003	up
MYOZ2	-8.233	<0.001	down
TPI1	-1.198	0.031	down
HSPB6	-3.141	0.003	down
COX17	-1.347	0.009	down
HSPB8	-2.592	0.009	down
LDHA	-2.170	0.004	down
EGR1	-3.265	<0.001	down
JUNB	-2.127	<0.001	down
LDB3	-6.778	<0.001	down
KIF1C	-1.094	0.033	down
ZFP36	-1.545	0.001	down
HSPB7	-8.114	<0.001	down
MYBPC1	-16.154	<0.001	down
ACKR3	1.053	0.040	up
PYGM	-8.684	<0.001	down
PPP1R27	-12.336	<0.001	down
VDAC3	-1.545	0.011	down
EIF3M	-1.013	<0.001	down
BIN1	-2.780	0.006	down
PTI	1.816	0.011	up
CXCL3	-2.561	<0.001	down
SLC25A4	-2.588	<0.001	down
CFL2	-1.997	0.001	down
GYG1	-1.439	0.012	down
ENO3	-5.748	<0.001	down
APOBEC2	-7.273	<0.001	down
MRPL33	-1.370	0.015	down
CASQ1	-7.564	<0.001	down
PRSS23	-1.387	0.004	down
SPP1	2.859	0.004	up
TMOD4	-7.526	<0.001	down
SLN	-14.307	<0.001	down
NDUFA4	-1.204	0.024	down
MSRB1	-1.859	0.004	down
FUNDC2	-1.044	0.018	down
SMPX	-13.923	<0.001	down
CYB5R1	-2.761	<0.001	down

NDUFV2	-1.164	0.022	down
MACROD1	-3.842	<0.001	down
SYNGR2	-1.679	0.004	down
CD276	1.213	0.012	up
UGP2	-1.026	0.010	down
ARPP19	-1.114	0.008	down
RYR1	-8.249	<0.001	down
IER2	-1.253	0.021	down
NMRK2	-12.813	0.001	down
PGM1	-1.694	0.004	down
ACTC1	-12.061	<0.001	down
ZFAND5	-1.193	0.038	down
CCN1	-1.722	<0.001	down
GOT2	-2.222	0.001	down
PALMD	-1.449	0.003	down
ATP2A2	-2.174	0.001	down

FC: fold change; CON: healthy cows; SCK: subclinical ketosis.

antioxidants

MDPI

Table S6. Differential lipid species in the serum samples between CON and SCK cows.

Lipid species	VIP	*P*-value	Log2FC(SCK/CON)	Regulate
Cer(d16:0/18:0)	4.665	0.019	0.261	Up
Cer(d24:0/18:0)	4.250	0.004	0.240	Up
Cer(d18:1/22:0)	4.298	0.020	0.174	Up
Cer(t18:0/18:0)	4.429	0.008	0.168	Up
SM(t18:1/14:0)	4.265	0.015	0.166	Up
Cer(d18:0/24:0)	1.631	0.046	0.148	Up
Cer(d18:1/24:0)	3.939	0.016	0.143	Up
Cer(d18:0/22:0)	1.467	0.029	0.136	Up
SM(d18:0/18:0)	1.567	0.046	0.135	Up
SM(d20:0/24:2)	1.761	0.038	0.135	Up
SM(d18:0/16:0)	1.587	0.031	0.119	Up
SM(d16:1/12:0)	2.113	0.020	0.109	Up
SM(d20:0/24:3)	1.954	0.015	0.107	Up
PC(19:1/18:2)	3.768	0.012	0.105	Up
Cer(d18:0/23:0)	1.843	0.026	0.096	Up
SM(d18:2/20:5)	1.884	0.029	0.084	Up
SM(t18:1/12:0)	1.904	0.038	0.082	Up
SM(d18:1/13:0)	2.073	0.036	0.077	Up
MGMG(39:0)	1.574	0.044	0.067	Up
LdMePE(18:2)	1.991	0.024	0.053	Up
MLCL(14:2/18:2/18:2)	2.054	0.015	0.045	Up
LPC(18:2)	2.419	0.024	0.035	Up
MLCL(18:2/14:1/18:1)	1.688	0.032	0.029	Up
LPC(18:1)	2.189	0.020	0.029	Up
PC(18:1/22:6)	1.746	0.050	0.028	Up
CL(18:2/16:0/16:0/24:1)	1.275	0.043	0.024	Up
SM(d17:1/18:1)	1.271	0.042	-0.016	Down
TG(18:4/16:0/16:0)	1.311	0.027	-0.017	Down
SM(d17:0/16:0)	1.513	0.034	-0.018	Down
PE(18:1p/20:4)	1.355	0.033	-0.020	Down
TG(16:0/16:1/16:1)	1.473	0.035	-0.021	Down
Hex1Cer(d24:1/18:0)	1.268	0.004	-0.021	Down
PE(18:0/18:1)	1.214	0.016	-0.022	Down
PE(16:0/18:1)	1.120	0.027	-0.023	Down
PC(8:0/11:2)	1.428	0.025	-0.024	Down
PC(18:3e/17:0)	1.712	0.023	-0.025	Down
CL(18:2/18:0/18:1/20:0)	1.542	0.007	-0.026	Down
PC(8:0/11:1)	1.687	0.015	-0.026	Down
CL(22:3/18:0/18:0/20:4)	1.529	0.039	-0.026	Down
PC(16:1e/17:0)	1.183	0.048	-0.028	Down
PC(32:1/20:4)	1.439	0.048	-0.028	Down

PC(11:0/22:4)	1.601	0.041	-0.028	Down
PE(18:0e/18:2)	1.078	0.032	-0.029	Down
CL(18:2/16:0/16:0/20:1)	1.330	0.048	-0.029	Down
SM(t18:1/21:4)	1.799	0.022	-0.029	Down
ChE(20:3)	1.859	0.014	-0.029	Down
SiE(18:3)	1.859	0.014	-0.029	Down
MePC(12:1e/23:0)	1.776	0.016	-0.030	Down
PC(18:0e/20:4)	1.776	0.016	-0.030	Down
SPH(t16:0)	1.610	0.036	-0.031	Down
CL(18:1/18:0/18:0/20:0)	1.537	0.002	-0.032	Down
PS(20:0e/18:2)	1.308	0.022	-0.032	Down
CL(22:3/16:0/16:0/20:4)	1.768	0.008	-0.034	Down
PC(31:1/20:4)	1.615	0.013	-0.036	Down
PE(16:0/20:4)	1.837	0.006	-0.037	Down
MePC(15:0/10:3)	2.251	0.036	-0.043	Down
PC(4:0/13:0)	2.169	0.002	-0.043	Down
PE(16:0/18:3)	1.181	0.023	-0.045	Down
TG(16:0/18:3/18:3)	1.825	0.032	-0.048	Down
PE(16:0/20:5)	1.510	0.025	-0.048	Down
PS(20:1e/20:4)	1.688	0.010	-0.050	Down
LPE(22:4)	1.452	0.040	-0.050	Down
PS(20:0e/20:4)	2.097	0.013	-0.051	Down
MePC(18:1e/18:2)	2.191	0.033	-0.052	Down
SM(d18:1/24:3)	2.424	0.005	-0.054	Down
PC(20:4e/19:1)	2.539	0.015	-0.055	Down
ChE(22:4)	2.608	0.002	-0.057	Down
dMePE(16:0/20:5)	1.823	0.029	-0.058	Down
TG(16:2e/10:4/18:0)	2.155	0.036	-0.058	Down
PE(16:1e/22:4)	1.757	0.008	-0.060	Down
PE(18:0/22:4)	1.971	0.008	-0.061	Down
PE(16:1e/18:2)	1.657	0.011	-0.063	Down
FA(20:4)	2.461	0.004	-0.066	Down
PS(18:0e/22:4)	1.826	0.045	-0.072	Down
PS(20:0e/22:4)	2.332	0.007	-0.083	Down
CL(18:3/18:0/18:2/20:0)	2.045	0.025	-0.104	Down
BisMePA(20:1/20:2)	3.660	0.023	-0.145	Down

VIP: variable importance in the projection; FC: fold change; CON: healthy cows; SCK: subclinical ketosis.

antioxidants MDPI

Table S7. Differential lipid species in the adipose tissue samples between CON and SCK cows.

Lipid species	VIP	*P*-value	Log2FC(SCK/CON)	Regulate
Cer(d18:2/20:0)	3.425	0.013	0.217	Up
SM(d18:1/16:0)	3.504	0.020	0.175	Up
Cer(t18:0/18:0)	4.477	0.011	0.162	Up
Cer(d18:1/24:0)	4.229	0.016	0.156	Up
SM(d18:1/24:0)	3.690	0.039	0.142	Up
TG(10:0/23:0/24:0)	2.367	0.044	0.133	Up
SM(d18:1/22:0)	3.605	0.017	0.111	Up
Cer(d17:0/16:0)	1.869	0.046	0.109	Up
Cer(d18:1/17:0)	1.463	0.041	0.089	Up
CL(18:2/16:0/16:0/22:1)	2.013	0.002	0.089	Up
SM(d18:0/16:0)	2.976	0.021	0.080	Up
SM(d18:1/18:3)	2.376	0.031	0.079	Up
PE(17:0/18:2)	1.672	0.006	0.074	Up
Cer(d18:1/16:0)	2.232	0.048	0.073	Up
Cer(d16:0/12:0)	3.205	0.020	0.058	Up
TG(16:0/14:1/20:4)	1.831	0.039	0.050	Up
Hex1Cer(d18:1/16:0)	1.759	0.003	0.048	Up
SM(d18:2/16:0)	1.909	0.046	0.048	Up
SM(d18:1/20:3)	1.402	0.047	0.045	Up
TG(16:0/18:1/20:4)	1.788	0.040	0.045	Up
TG(18:4/14:0/18:1)	1.421	0.015	0.036	Up
MGDG(14:0e/16:0)	1.372	0.008	0.035	Up
PC(16:1/18:2)	1.356	0.035	0.034	Up
Cer(d16:0/18:0)	1.440	0.037	0.033	Up
TG(8:0/10:2/18:1)	1.483	0.021	0.030	Up
Cer(d16:0/18:0+O)	1.301	0.028	0.029	Up
Cer(t18:0/16:0)	1.301	0.028	0.029	Up
Cer(d16:0/16:0)	1.335	0.038	0.027	Up
MePC(12:1e/20:4)	1.387	0.042	0.026	Up
CL(18:2/18:0/18:1/20:0)	1.088	0.033	0.025	Up
PC(16:0/16:0)	1.199	0.033	0.024	Up
CL(18:1/18:0/18:0/20:0)	1.068	0.003	0.019	Up
CL(22:3/18:0/18:0/20:4)	1.010	0.028	0.017	Up
TG(18:3e/14:1/18:4)	1.115	0.003	0.014	Up
MePC(14:0e/18:2)	1.219	0.033	-0.036	Down
PC(14:0e/18:2)	1.235	0.039	-0.043	Down
Cer(d18:1/18:0)	1.516	0.045	-0.045	Down
PC(16:0/19:0)	2.550	0.018	-0.048	Down
PS(18:0e/18:1)	1.528	0.028	-0.050	Down
PC(17:0/20:4)	1.524	0.015	-0.051	Down
Cer(d15:0/26:2)	1.386	0.036	-0.052	Down

PE(18:0/22:5)	1.343	0.040	-0.053	Down
PC(16:2e/18:1)	1.954	0.045	-0.058	Down
MePC(16:0e/18:2)	1.750	0.009	-0.061	Down
PC(20:0/18:1)	1.750	0.019	-0.061	Down
MePC(15:0/18:1)	1.795	0.011	-0.061	Down
Cer(d16:0/21:1)	1.518	0.024	-0.066	Down
PE(16:0p/18:2)	1.752	0.010	-0.067	Down
PC(16:1e/23:0)	1.880	0.001	-0.074	Down
PC(11:0/20:2)	1.536	0.029	-0.075	Down
PC(18:1e/16:0)	2.616	0.000	-0.076	Down
PC(15:0/16:0)	2.189	0.019	-0.077	Down
PC(16:0/13:0)	2.249	0.022	-0.081	Down
PS(17:0/18:1)	2.015	0.032	-0.095	Down
PE(19:1/18:1)	2.326	0.037	-0.098	Down
PE(20:0/18:1)	2.836	0.009	-0.112	Down
PC(18:0e/22:5)	1.924	0.032	-0.120	Down
LPC(16:0e)	2.740	0.012	-0.121	Down
LPC(18:0e)	2.286	0.044	-0.123	Down
Hex1Cer(d18:1/22:0)	1.904	0.009	-0.128	Down
PC(18:1e/22:6)	2.009	0.049	-0.132	Down
PE(20:1e/20:4)	2.298	0.044	-0.134	Down
PE(20:4e/15:0)	3.125	0.002	-0.147	Down
TG(18:1e/14:0/16:0)	3.362	0.011	-0.156	Down
PC(18:3e/18:1)	2.315	0.005	-0.158	Down
LPC(18:1e)	2.741	0.018	-0.159	Down
PE(18:1p/20:1)	3.166	0.042	-0.161	Down
PI(18:0/20:2)	2.207	0.008	-0.167	Down
Hex1Cer(d18:1/24:0)	2.495	0.029	-0.171	Down
TG(18:2/17:1/18:2)	1.538	0.023	-0.197	Down
PE(18:2/18:2)	1.595	0.016	-0.199	Down
PE(18:1p/20:4)	3.407	0.020	-0.204	Down
PE(18:2e/20:4)	2.891	0.019	-0.208	Down
PE(20:4e/13:0)	3.582	0.005	-0.218	Down
PI(18:1/20:3)	2.629	0.002	-0.220	Down
Hex1Cer(d19:2/23:0+O)	3.562	0.027	-0.232	Down
PC(20:2/22:6)	2.423	0.024	-0.244	Down
PC(26:1/10:1)	4.391	0.000	-0.248	Down
Hex1Cer(t18:0/18:1)	3.169	0.010	-0.294	Down

VIP: variable importance in the projection; FC: fold change; CON: healthy cows; SCK: subclinical ketosis.

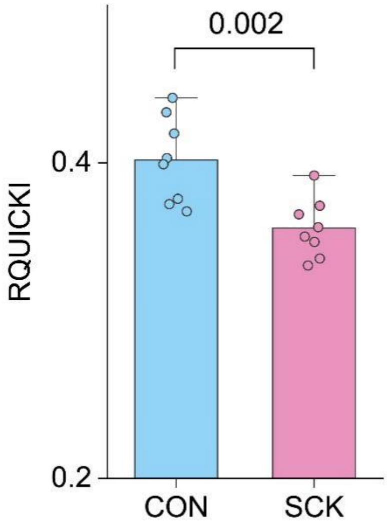

Figure S1. Revised quantitative insulin sensitivity check index (RQUICKI) of health cows and subclinical ketosis cows. CON: healthy cows; SCK: subclinical ketosis.

antioxidants MDPI

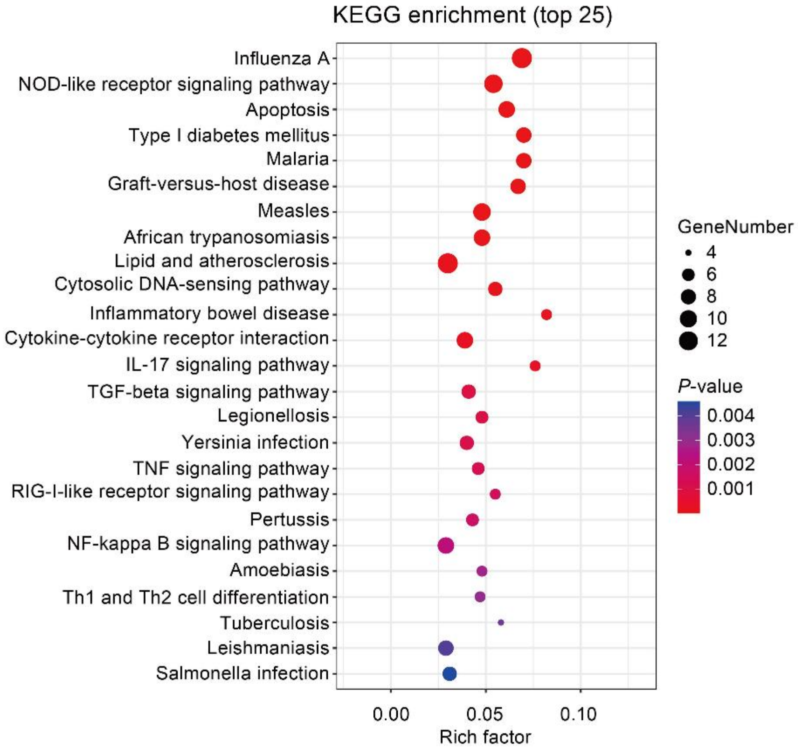

Figure S2. KEGG pathway enrichment analysis of whole blood (top 25 terms).

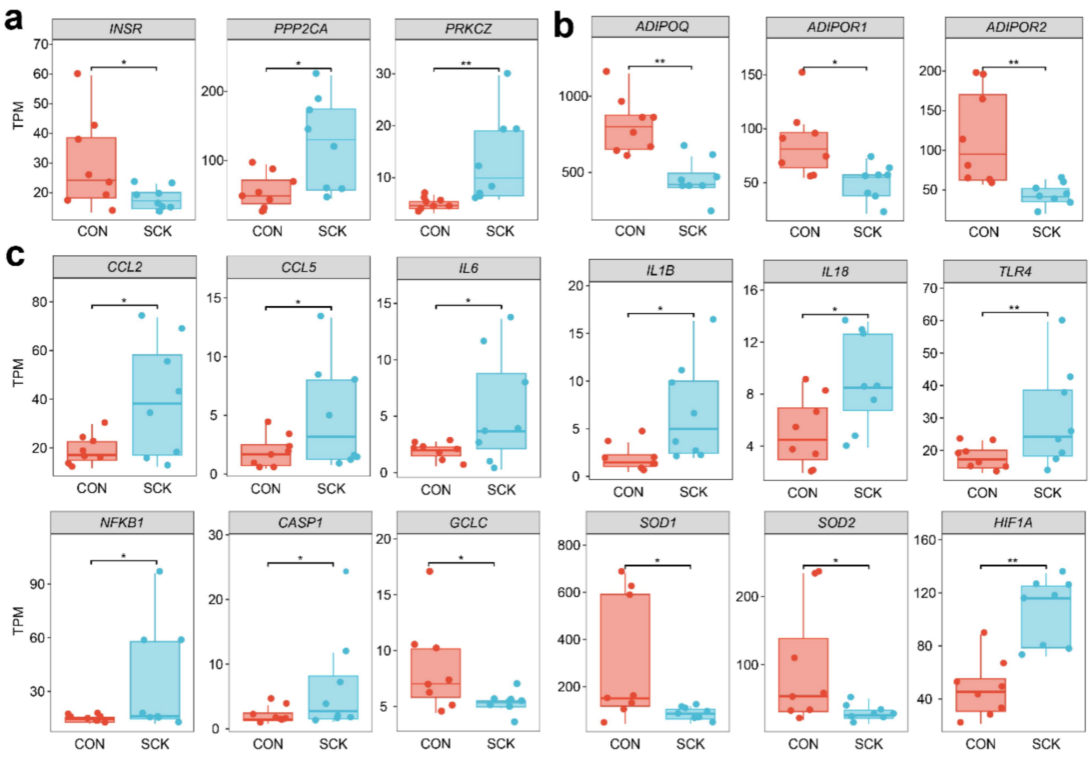

Figure S3. Representative differential genes in adipose tissue. Transcripts per million (TPM) of selected gene representatives of (**a**) insulin signaling pathway, (**b**) adiponectin production, and (**c**) inflammatory and oxidative response. DESeq2 package was used to identify the DEGs using a cutoff threshold of adj. $P < 0.05$ and fold change (FC) > 2. The asterisks indicate statistically significant differences and correspond to *$P < 0.05$, ** $P < 0.01$, and *** $P < 0.001$. CON: healthy cows; SCK: subclinical ketosis.

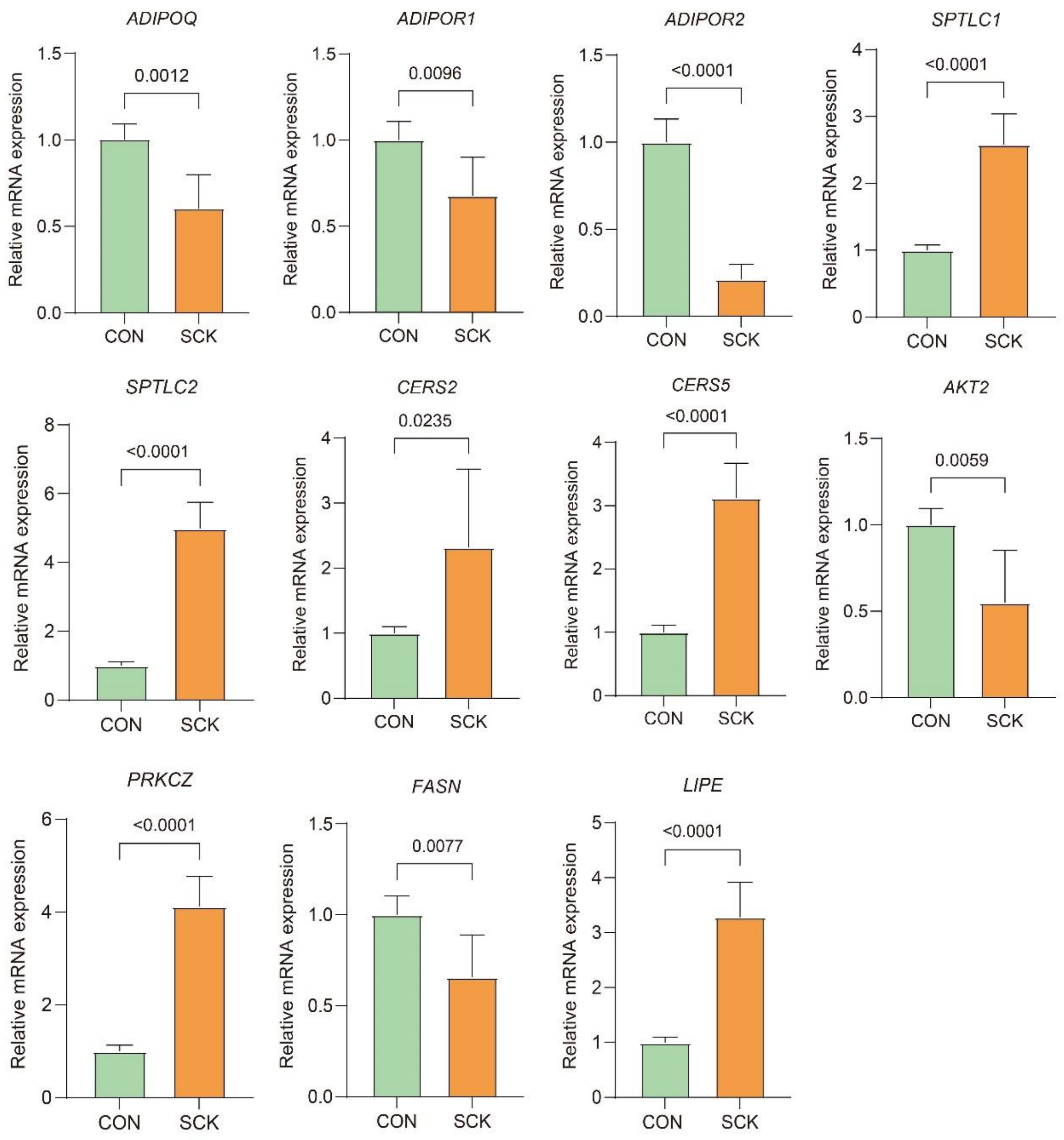

Figure S4. Validation of adipose tissue differential gene expression of RNA-seq by qPCR. CON: healthy cows; SCK: subclinical ketosis.

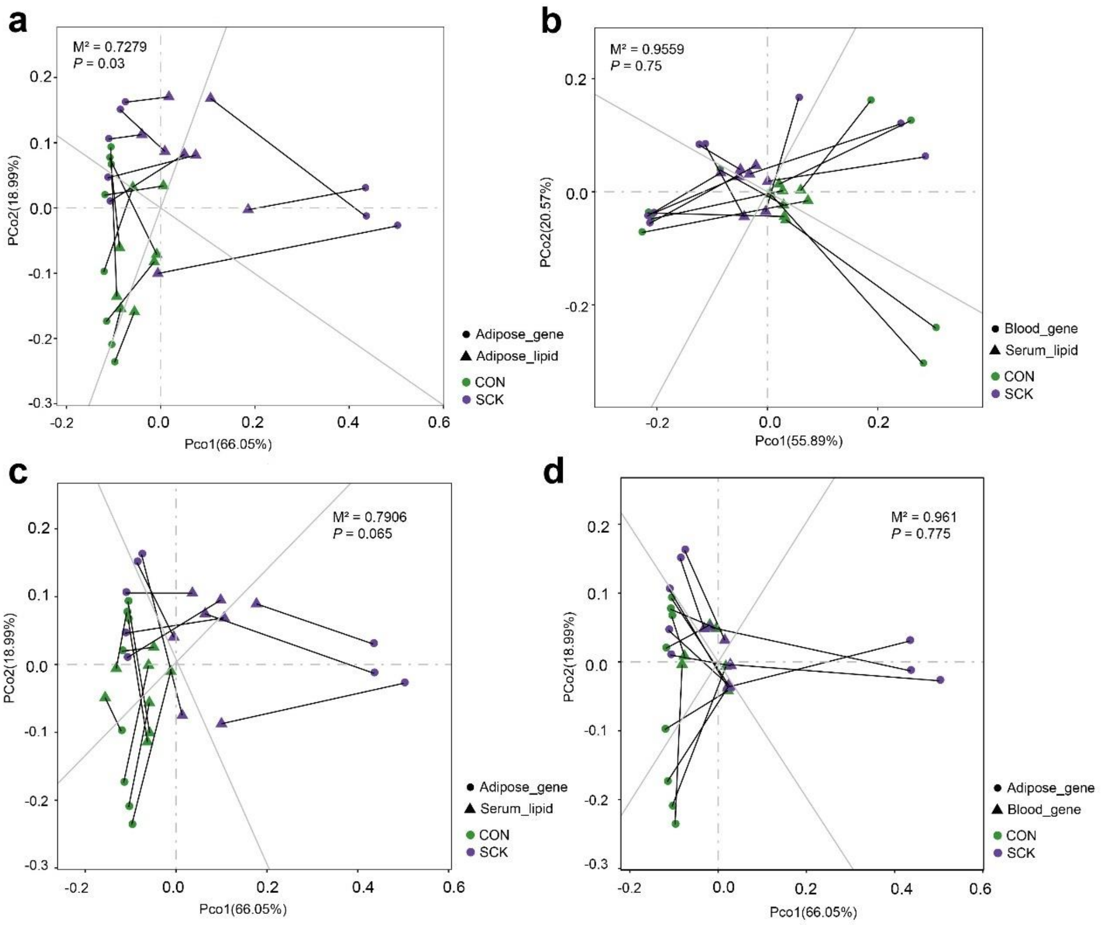

Figure S5. Procrustes analysis of the relationship between differential lipid features and differentially expressed gene (DEG). (**a**) Procrustes analysis of differential lipids and DEG in adipose tissue. (**b**) Procrustes analysis of serum differential lipids and blood DEG. (**c**) Procrustes analysis of serum differential lipids and DEG in adipose tissue. (**d**) Procrustes analysis of blood DEG and adipose tissue DEG. The correlation is considered significant when $P < 0.05$. CON: healthy cows; SCK: subclinical ketosis.

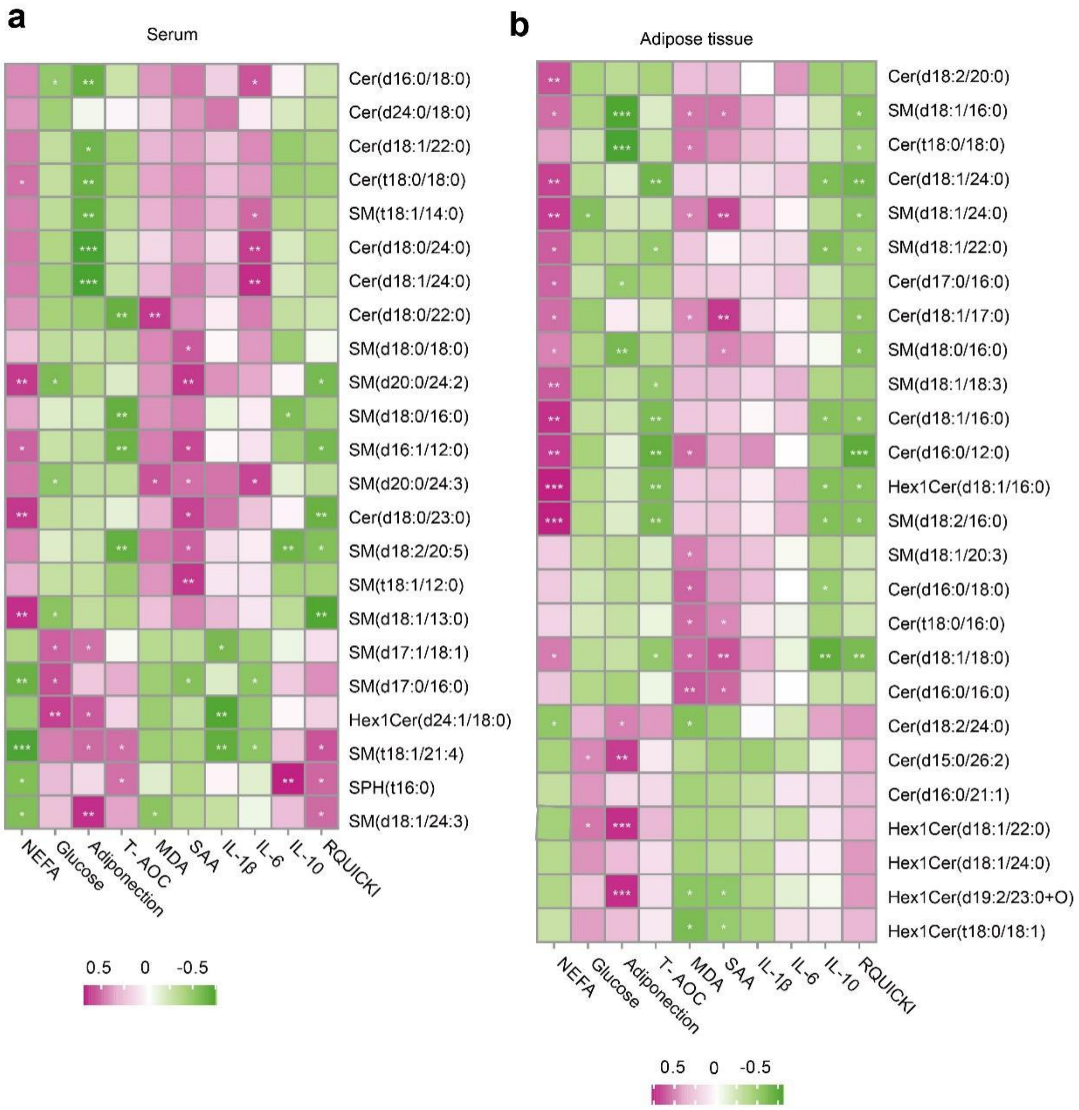

Figure S6. Heatmap of association between serum biochemical parameter and differential sphingolipids in serum (**a**) and adipose tissue (**b**). The asterisks indicate statistically significant differences and correspond to **P* < 0.05, ** *P* < 0.01, and *** *P* < 0.001. NEFA: nonesterified fatty acids; T-AOC: total antioxidant capacity; SOD: superoxide dismutase; MDA: malonaldehyde; MDA: malonaldehyde; RQUICKI: revised quantitative insulin sensitivity check index.
